# Reverse Weak Polarity‐Induced Ordered Layer Control for Enhanced Second‐Harmonic Generation in Ultraviolet Nonlinear Optical Crystals

**DOI:** 10.1002/advs.75642

**Published:** 2026-05-11

**Authors:** Lingli Wu, Chensheng Lin, Bing‐Xuan Li, Huixin Fan, Lingfei Lv, Shunda Yang, Tao Yan, Min Luo

**Affiliations:** ^1^ State Key Laboratory of Functional Crystals and Devices Fujian Institute of Research on the Structure of Matter Chinese Academy of Sciences Fuzhou Fujian China; ^2^ Fujian College University of Chinese Academy of Sciences Fuzhou Fujian China

**Keywords:** layered structures, polarity transfer, second harmonic generation, ultraviolet nonlinear optical crystal

## Abstract

There is an urgent need to achieve a large second‐harmonic generation (SHG) response in ultraviolet nonlinear optical (UV NLO) crystals to advance UV laser technologies. SHG efficiency depends on the alignment and properties of functional groups, particularly π‐conjugated units with high hyperpolarizability. Layered structures are widely recognized as an optimal template for densely preorganizing π‐conjugated units into orderly arrays, yet the interlayer linkers are typically metal cations that couple adjacent layers through largely non‐directional ionic interactions, often producing random even antiparallel stacking that cancels macroscopic SHG. Herein, we propose a *Reverse Weak Polarity‐Induced Ordered‐layer Control* (*RPIO*) strategy, in which a “layer→linker→layer” polarity‐transfer pathway—driven by dipole–dipole interactions—enforces consistent alignment of polar layers. Guided by this strategy, three isostructural compounds, RE(C_3_H_2_O_4_)NO_3_·4H_2_O (RE = Y, Gd, Lu), are synthesized, featuring polar ${\left[\text{RE}\left(C_{3}H_{2}O_{4}\right){\left(H_{2}O\right)}_{4}\right]}_{\infty }^{+}$ layers coherently aligned induced by NO_3_^−^ linkers. All compounds exhibit large SHG responses (8.5–9.5× KDP), wide band gaps (∼4.13 eV), sizable birefringences (∼0.135 at 589.3 nm), and favorable growth habits. By explicitly engineering interlayer connectivity, this work establishes a predictable and controllable route to coherently stack polar layers and thereby maximize SHG in layered UV NLO materials.

## Graphical abstract

*Reverse Weak Polarity‐Induced Ordered Layer Control* strategy, optimizing the design of layered structures, has been successfully designed. By leveraging inhomogeneous field of units to replace isotropic fields of metal cations, polar layers realize controllable layer stacking via a “layer→linker→layer” polarity transfer. Ultimately, we successfully design three promising ultraviolet nonlinear optical crystals, RE(C_3_H_2_O_4_)NO_3_·4H_2_O (RE = Y, Gd, Lu).

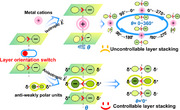

## 1 Introduction

Ultraviolet nonlinear optical (UV NLO) crystals are indispensable for solid‐state frequency conversion to generate UV laser outputs and thereby support modern optoelectronic technologies [1, 2, 3, 4, 5]. A central performance target for UV NLO crystals is the large efficiency of second‐harmonic generation (SHG), which is primarily governed by three factors according to the anionic group theory: the inherent properties of the functional groups, their structural alignment, and density [6, 7]. Accordingly, high‐performance UV NLO crystals typically incorporate π‐conjugated groups (e.g., BO_3_^3−^, NO_3_^−^, CO_3_^2−^, B_3_O_6_^3−^, C_3_N_3_O_3_^3−^, C(NH_2_)_3_^+^) with large hyperpolarizability (*β*), where delocalized π‐electrons enable pronounced asymmetric polarization under an external electro‐optic field [8, 9, 10, 11, 12]. Therefore, the coherent and dense alignment of these functional groups becomes a key factor in the design of efficient crystal structures.

**FIGURE 1:**
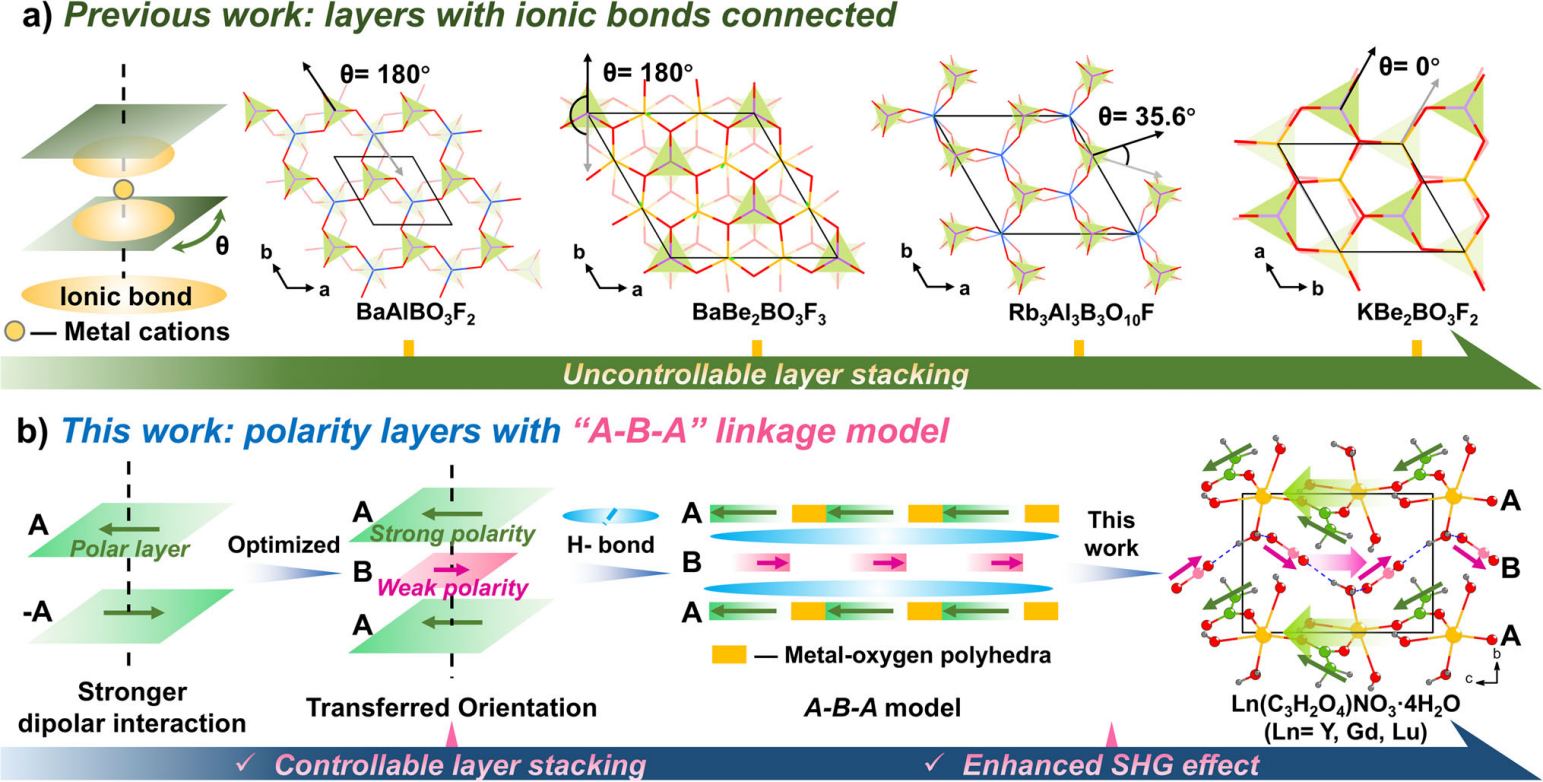
(a) The traditional layered structures rely on metal cations to connect the layers, resulting in uncontrollable layer stacking; (b) the *RPIO* strategy and “A‐B‐A” linkage model are proposed to realize controllable layer stacking and enhance SHG effect.

Layered structures are widely regarded as an ideal design paradigm for UV NLO crystal design because they can densely preorganize π‐conjugated units into orderly 2D planar arrays [1, 13]. In typical layered structures of UV NLO materials, π‐conjugated groups are connected by metal–oxygen polyhedra to form 2D layers, while adjacent layers are coupled by interlayer metal cations through predominantly ionic interactions. Representative systems, such as [Be_2_BO_3_F_2_]_∞_ (KBBF) [14], [Li_2_M_4_B_6_O_20_F]_∞_ (A_3_Ba_3_Li_2_M_4_B_6_O_20_F; A = K, Rb; M = Al, Ga) [15, 16, 17], [Zn_2_BO_3_X]_∞_ (AZn_2_BO_3_X; A = Na, K, Rb, NH_4_; X = Cl, Br) [18, 19, 20], and [Zn_2_(CO_3_)_2_(OH)_2_]_∞_ (NaZnCO_3_(OH)) [21], demonstrate the effectiveness of this construction paradigm. However, precise control over interlayer orientation remains a persistent bottleneck. Thermodynamically, the Gibbs free energy minimization principle favors centrosymmetric (CS) structures, which tend to antiparallel stacking of polar layers [22, 23, 24], Structurally, the intrinsically non‐directional nature of ionic bonding weakens interlayer correlation, leading to random stacking and making rational predesign difficult (as shown in Figure 1a) [14, 25, 26, 27]. Importantly, the stacking geometry of the layers directly dictates SHG performance—fully antiparallel alignment (e.g., BaAlBO_3_F_2_) [25] yields CS structures with no SHG signal, while partial antiparallel alignment (e.g., Rb_3_Al_3_B_3_O_10_F) [27] limits SHG maximization. Thus, optimizing single‐layer configurations without addressing systematic interlayer interactions hinders full SHG enhancement.

To overcome this, we propose an interlayer engineering concept in which specific functional groups are deliberately introduced as linkers to establish a “layer→linker→layer” polarity‐transfer pathway through dipole‐dipole interactions, thereby enforcing consistent layer orientation. Specifically, we propose a *Reverse Weak Polarity‐Induced Ordered Layer Control* (*RPIO*) strategy, wherein weakly polar structural units are “reversed” into the target polar layers, creating localized anti‐polar “leads” that drive the parallel alignment of dipole moments between adjacent polar layers, thereby enabling precise regulation of layer alignment (Figure 1b). For practical implementation, we introduce an “A‐B‐A” linkage model with two design key points: (1) A‐layers contain strongly polar functional groups, while B‐linkers employ weakly polar groups with reverse orientation, enabling robust polarity contrast and facilitating polar structure formation; (2) To achieve A–B coupling, directional interactions—beyond the limitation of non‐directional ionic coupling—must be leveraged. Thus, hydrogen (H‐) bonding with deliberate donor–acceptor (D–A) pairing is employed to strengthen interlayer correlation and reduce stacking randomness [28, 29]. Explicitly, the A‐B‐A model features A‐layers incorporating strongly polar units (with interlayer H‐donors/acceptors), and B‐linkers comprising weakly reverse‐polar groups (with complementary H‐acceptors/donors), collectively enabling polarity transfer for coherent layer alignment.

To validate the A‐B‐A model and maximize SHG performance, we select the π‐conjugated C_3_H_2_O_4_^2−^ unit, which exhibits significant polarizability anisotropy and hyperpolarizability due to its asymmetric electron density distribution and strong electronic delocalization [30]. Lanthanide ions RE^3+^ (Y^3+^, Gd^3+^, Lu^3+^) are chosen as the metal nodes to construct the layers with C_3_H_2_O_4_^2−^ units, based on three key considerations: (1) Their equatorial coordination plane supports layered formation with C_3_H_2_O_4_^2−^ units adopting a 1‐bidentate‐2‐monodentate coordination mode, similar to CO_3_^2−^ in ${\left[{\text{MgCO}}_{3}F\right]}_{\infty }^{-}$ and BO_3_^3−^ in ${\left[{\text{MgBO}}_{3}F\right]}_{\infty }^{2-}$ [31, 32, 33, 34] (2) their large ionic radii and high charge facilitate additional coordination sites for OH^−^ or H_2_O molecules in aqueous/hydrothermal conditions (e.g., RE_5_(C_3_N_3_O_3_)(OH)_12_ (RE = Y, Yb, Lu) [35], RE(OH)_2_NO_3_ (RE = La, Gd, Y) [36], Na_3_Y(CO_3_)_3_·3H_2_O [37]), which can serve as H‐donors for the B‐linkers; (3) The irregular coordination of RE‐oxygen polyhedra contributes to the SHG effect, and RE^3+^ ions (Y^3+^/ Lu^3+^ with closed‐shell configurations and Gd^3+^ with a half‐filled stable state) do not narrow the bandgap, making them suitable for UV applications [38]. For the B‐linkers, NO_3_^−^ groups are selected to satisfy charge balance and function as H‐acceptors. Although NO_3_^−^ group is nominally rigid and nonpolar, limited distortion in coordination environments can render it weakly polar, as exemplified by Bi_3_TeO_6_OH(NO_3_)_2_ [39], A_2_Hg(NO_3_)_4_ (A = K, Rb) [40], and (C_2_H_5_N_4_)(NO_3_) [41]. Thus, NO_3_^−^ units can generate weak reverse dipole moments under the effect of the polar A‐layers, fulfilling the B‐linker requirement. Additionally, the π‐conjugated nature of NO_3_^−^ may contribute to the SHG effect, and its planar structure can reduce the interlayer spacing of the A‐layers, thereby increasing the density of all functional groups.

Ultimately, three isostructural compounds RE(C_3_H_2_O_4_)NO_3_·4H_2_O (RE = Y, Gd, Lu) have been successfully designed and synthesized. As expected, the C_3_H_2_O_4_^2−^ groups coordinate with RE^3+^ (Y^3+^, Gd^3+^, Lu^3+^) in the 1‐bidentate‐2‐monodentate mode to form highly polar A‐layers, ${\left[\text{RE}\left(C_{3}H_{2}O_{4}\right){\left(H_{2}O\right)}_{4}\right]}_{\infty }^{+}$, where the remaining coordination sites of RE^3+^ are occupied by H_2_O molecules acting as H‐donors. The NO_3_^−^ units served as B‐linkers and H‐acceptors couple with coordinated H_2_O molecules, forming the thin pseudo‐layer ${\left[{\text{NO}}_{3}\left(H_{2}O\right)\right]}_{\infty }^{-}$ in the same mode of 1‐bidentate‐2‐monodentate. Through control layer alignment, C_3_H_2_O_4_^2−^ and NO_3_^−^ groups achieve uniform arrangements, endowing RE(C_3_H_2_O_4_)NO_3_·4H_2_O with exceptionally large SHG effects, experimentally measured as 8.5–9.5× KDP. Additionally, these compounds all exhibit wide bandgaps of ∼4.13 eV, sizeable birefringences of ∼0.135 @589.3 nm, and good growth habits. This strategy provides a comprehensive principle for achieving consistent alignment in highly polar layered structures and demonstrates the potential for UV applications with the synthesis of three novel crystals that exhibit significant SHG effects.

## 2 Result and Discussion

**FIGURE 2:**
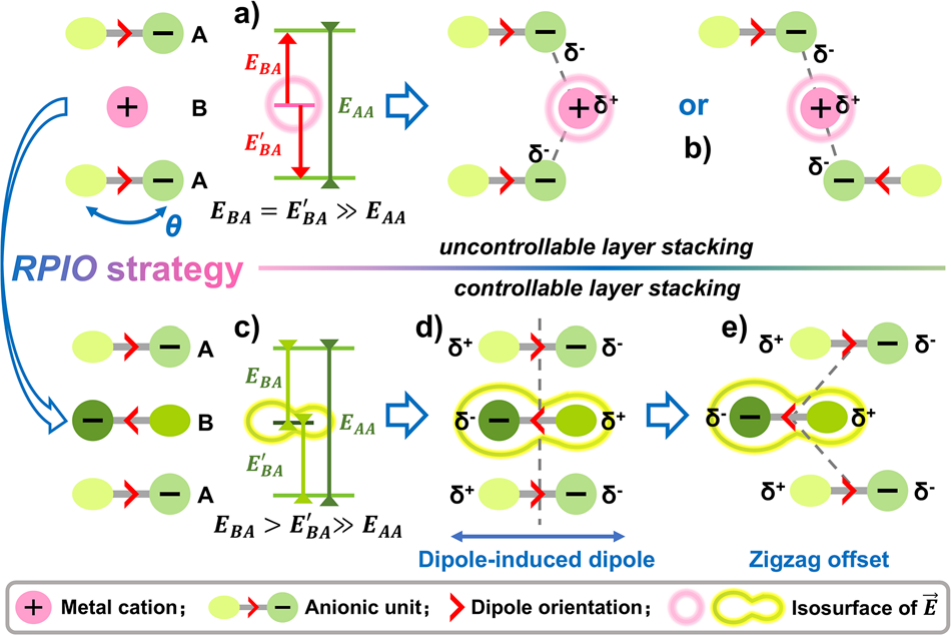
(a, c) Interlayer electric field differs between traditional layered structures and *RPIO*‐designed layered structures; (b) random layer stacking in traditional layered structures; (d) through dipole‐induced dipole, realizing dipole transfer; (e) zigzag offset of units with the same electronegativity.

The essence of regulating layer orientation in layered structures lies in the coupling between electric field forces and interlayer interactions. According to Coulomb's law, the electric field *E* is inversely proportional to the square of distance; thus, in layered structures, interlayer linkers dominate the orientation of layers, while the electric field effect between layers is much weak (i.e. ***E***_*layer* − *layer*_ ≪ ***E***_*linker* − *layer*_) [42]. In traditional layered structures, metal cations act as interlayer linkers, connecting anionic layers through ionic bonds. However, metal cations can be likened to uniformly charged bodies, exhibiting isotropic Coulombic fields in space, with no directional preference in attracting anions (e.g. $E_{BA}=E_{BA}^{\prime }$ in Figure 2a). Consequently, the absence of directional forces between anionic layers leads to randomized layer orientation, making ordered stacking challenging (Figure 2b). In contrast, the *RPIO* strategy is proposed to address this challenge—introducing an anti‐weakly polar anionic unit as a “lead” within the anionic layers. By using functional groups as interlayer linkers, the electronegativity difference within the groups forms an inherent potential difference, generating an endogenous inhomogeneous electric field (e.g. $E_{BA}>E_{BA}^{\prime }$ in Figure 2c). This electric field induces polar groups in adjacent layers to adjust their orientation, thereby reducing system energy. In this work, we introduced the non‐polar NO_3_^−^ group (Figure 2d). The strong electric field generated by the interlayer anionic C_3_H_2_O_4_^2−^ groups deforms the electron cloud of the NO_3_^−^ group to induced dipoles. Thus, the NO_3_^−^ groups establish the pathway for polar transmission, enabling the uniform alignment of C_3_H_2_O_4_^2−^‐layers. Moreover, for groups with the same electronegativity, intense Coulombic interactions may force the anionic groups into a zigzag arrangement (Figure 2e). Ultimately, guided by this strategy, we synthesized three compounds—RE(C_3_H_2_O_4_)NO_3_·4H_2_O (RE = Y, Gd, Lu) (abbreviated as YCN, GCN, and LCN).

YCN, GCN, and LCN were all prepared by the aqueous solution method, and large‐sized single crystals were obtained through slow evaporation. Since these crystals are all isostructural and belong to the *Pmn*2_1_ space group, the morphologies of these crystals are similar (Figure S1). Crystals of YCN, GCN, and LCN all maintained good stability when exposed to the air for six months. Energy Dispersive X‐ray Spectroscopy analysis (EDS) and the Powder X‐ray diffraction (XRD) patterns confirmed that the obtained samples contain the target elements and exhibit pure phases (Figures S2 and S3). The decomposition temperatures of this series of crystals were approximately 125°C as determined by the thermogravimetric and differential scanning calorimetry (TG‐DTA) detection (Figure S4).

**FIGURE 3:**
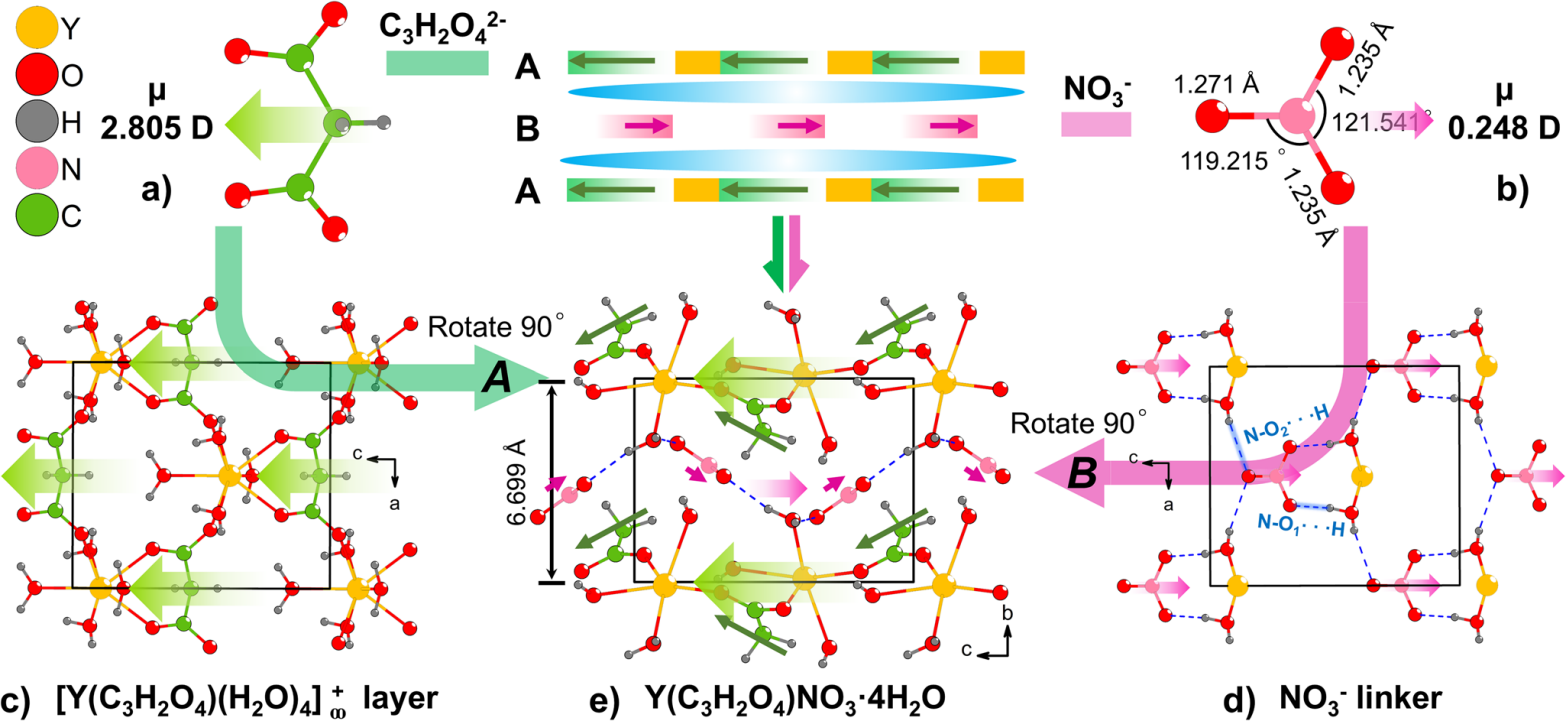
(a, b) The states of C_3_H_2_O_4_^2−^ and NO_3_^−^ groups in YCN; (c) ${\left[Y\left(C_{3}H_{2}O_{4}\right){\left(H_{2}O\right)}_{4}\right]}_{\infty }^{+}$ layer; (d) H‐bond network of NO_3_^−^ linker; (e) structure of YCN.

As three compounds are isostructural, YCN serves as a representation for discussing their structures. YCN exhibits a layered structure, where strongly polar A‐layers and weakly polar B‐linkers are alternately stacked, forming a sandwich‐like “*A‐B‐A”* configuration. (1) A‐layers structure: The highly polar layers ${\left[Y\left(C_{3}H_{2}O_{4}\right){\left(H_{2}O\right)}_{4}\right]}_{\infty }^{+}$ are assembled by C_3_H_2_O_4_^2−^ and Y^3+^ via a coordination mode of one bidentate and two monodentate ligands (Figure 3c). This unique arrangement ensures the uniform alignment of C_3_H_2_O_4_^2−^ within the layer, with its dipole moment (µ = 2.805 D) oriented along the *c*‐axis (Figure 3a). Y^3+^ adopts an eight‐coordination geometry, with Y─O bond lengths spanning 2.290–2.418 Å, while the remaining coordination sites are occupied by H_2_O molecules. (2) B‐linkers structure: The weakly polar linkers NO_3_^−^ are constructed by the H_2_O molecules from neighboring A‐layers through hydrogen bonds, adopting the same bidentate‐monodentate coordination mode like A layers (Figure 3d). Such a connection mode also ensures that NO_3_^−^ units are arranged uniformly and nearly parallel to A layers with a short layer spacing of 6.699 Å. Although NO_3_^−^ unit is inherently nonpolar, it undergoes slight distortion within the coordination environment (N─O bond lengths: 1.271‐1.235 Å; O─N─O bond angles: 119.215°–121.541°), reducing from *D*3*h* symmetry to *C*2*v*, and exhibiting a small dipole moment (µ = 0.248 D) opposite the *c*‐axis (Figure 3b). The significant difference in the dipole moment between C_3_H_2_O_4_^2−^ and NO_3_^−^ makes YCN crystallize in polar space group (Figure 3e). Furthermore, the slight distortion of NO_3_^−^ confirms rigid units only retain weak polarity upon deformation, which could provide guidance for selecting suitable B‐linkers for A‐B‐A models in future material engineering.

Due to the cost advantage of YCN, this study focuses on the controlled crystal growth of YCN, yielding high‐quality single crystals with dimensions up to 14 × 14 × 6 mm^3^. The maximum growth planes of YCN crystals were confirmed by XRD patterns to be (010), (101), and (011) facets, consist with the simulation diagram (Figure S5). During the crystal growth, no layered growth behavior was observed. We use the first‐principles method, symmetry‐adapted perturbation theory (SAPT), to decompose and characterize the interlayer interactions [43]. Since layers are connected via H bonds between NO_3_^−^ and H_2_O molecules (as shown in Figure 3d, there are two types of H bonds: N─O_1_···H and N─O_2_···H), corresponding calculations were performed for these relative fragments. Table S6 demonstrated that electrostatic energy dominates the attractive forces, with the total energy exhibiting stronger net attractive effects [44]. This is because the charged groups NO_3_^−^ possess stronger attraction capabilities than neutral units. Considering the binding mode of NO_3_^−^ units with the H_2_O fragments in Layers, the doubled superposition of charge‐assisted N─O_1_···H and N─O_2_···H interaction energies can reach considerable intensity in connecting layers, thereby effectively suppressing crystal delamination.

The reflectance and transmission spectrums of YCN, GCN, LCN shown that the cutoff edges are ∼300 nm, matching the optical band gaps of ∼4.13 eV (Figure S6). The cutoff edges at approximately 300 nm are presumed to arise primarily from the intrinsic electronic transition of NO_3_^−^ groups. Similar absorption edge characteristics are also observed in crystals such as K_3_B_6_O_10_NO_3_ [45], KNO_3_SO_3_NH_3_ [46], and (C_2_H_5_N_4_)(NO_3_) [41]. Subsequently, the GGA functional was employed to calculate the band gaps, obtaining values of 3.75, 3.68, and 3.68 eV, respectively, with scissor operator corrections around 0.4 eV (Figure S7). Furthermore, the partial density of states (PDOS) spectrums for YCN, GCN, and LCN, as illustrated in Figure S8, demonstrate that the valence band maximum (VBM) is predominantly composed of O‐2p states, whereas the conduction band minimum (CBM) arises primarily from contributions of O‐2p and N‐2p orbitals. Consequently, the bandgaps are determined by NO_3_^−^ units.

**FIGURE 4:**
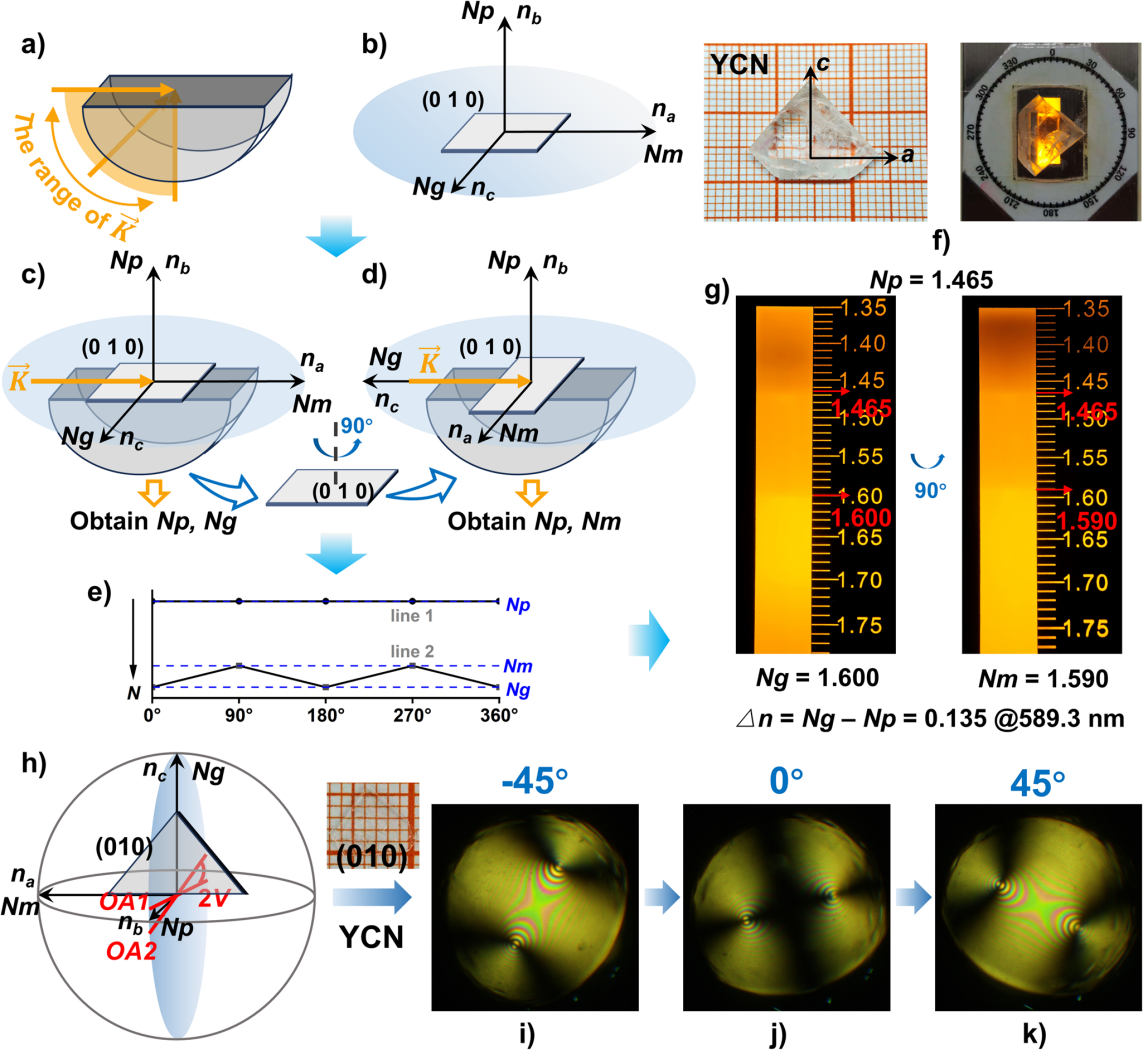
(a) The semicircular prism with incident light $\vec{K}$ along the prism's radius; (b) The refractive index ellipsoid of YCN, GCN, and LCN; (c, d) The crystal plane is fitted to the semicircular prism, and one of the incident lights that nearly parallel to the long side of the prism can measure the extreme refractive index in corresponding position; (e) estimated test results for (010) facet with two lines; (f) the single crystal photo of YCN and its (010) facet; (g) the corresponding experimental refractive indices for YCN; (h) the positions of two optic axes in refractive index ellipsoid; (i‐k) the conoscopic interference patterns of (010) facets for YCN.

Since suitable size of crystals had been obtained, refractive indices can be characterized through the immersion technique using an FGE‐002A Gem refractometer with 589.3 nm light (Figure 4a) [47, 48]. The calculated birefringence of YCN was 0.158 @589.3 nm, with the refractive indexes following the relationship of *n_c_* > *n_a_* > *n_b_* (Figure S9a). To experimentally verify this, a (010) crystal facet was prepared to measure the refractive index *Ni* (*i* = *g*, *m*, *p*) along the principal optical axes, corresponding to *n_c_*, *n_a_*, and *n_b_* in the crystallographic axes (Figure 4b). By analyzing the relationship between the incident light wave and the refractive index ellipsoid, after rotating the facet above the prism, two distinct light‐dark lines can be clearly seen within the scale (Figure 4c–e). Line 1 represents the refractive index *Np*, while line 2 shows the refractive index variation ranging from *Nm* to *Ng*. Specifically, the values of *Ng* and *Nm* appear at the position where the *a*‐axis is parallel and perpendicular to the long edge of the prism. During the testing process, the results matched expectations: Line 1 remained nearly unchanged, while Line 2 exhibited extreme values at the specified positions (Figure 4f,g). Therefore, the refractive indices of YCN at 589.3 nm are *Ng* = 1.600, *Nm* = 1.590, *Np* = 1.465, yielding a birefringence of 0.135 @589.3 nm, consistent with the calculated value. Similarly, through the same testing method, the experimental birefringence values for GCN and LCN are 0.134 and 135 @589.3 nm, matching the calculated values of 0.155 and 0.158 @589.3 nm, respectively (Figures S9b,c and S10).

Additionally, the close values of *Ng* and *Nm* suggest small optic axial angle (2*V*) values of YCN, GCN, and LCN, which can further be derived to be 29.64°, 29.76°, and 31.14° using Equation S1, respectively. Furthermore, conoscopic interference experiments were also employed to estimate the 2*V* angle [49]. As for YCN, GCN, and LCN are the negative biaxial optical crystals (i.e. *Ng*‐ *Nm*< *Nm*‐ *Np*), two optic axes (*OA1* and *OA2*) are close to *Np* (*n_b_*); thus, the (010) facets were conduct to measure the 2*V* angles (Figure 4h). The conoscopic interference pattern of (010) facets, observed at ± 45°, revealed the dark bands with curvature approaching 90°, confirming its minimal 2*V* angle (Figure 4i–k; Figure S11). Therefore, optical measurements also confirmed the close agreement between the refractive indices *Ng* and *Nm*. The larger birefringence values and the smaller 2*V* angles of YCN, GCN, and LCN can be explained through the electron density map (Figure S9d,e). Analysis revealed similar anisotropic electron cloud distributions along the *a*‐ and *c*‐axes, consistent with their small 2*V* angles. Notably, electron density was localized within layers and NO_3_^−^ units, with negligible interlayer distribution. This pronounced anisotropy between intralayer (*a/c*) and interlayer (*b*‐axis) directions directly accounts for their large birefringences. Such sufficiently large birefringences can more easily satisfy the phase‐matching conditions, with wider angle and wavelength matching bandwidth. The calculated Type I phase‐matching wavelengths were all below 300 nm, ensuring that phase matching can be achieved within the transmission range (Figure S9).

**FIGURE 5:**
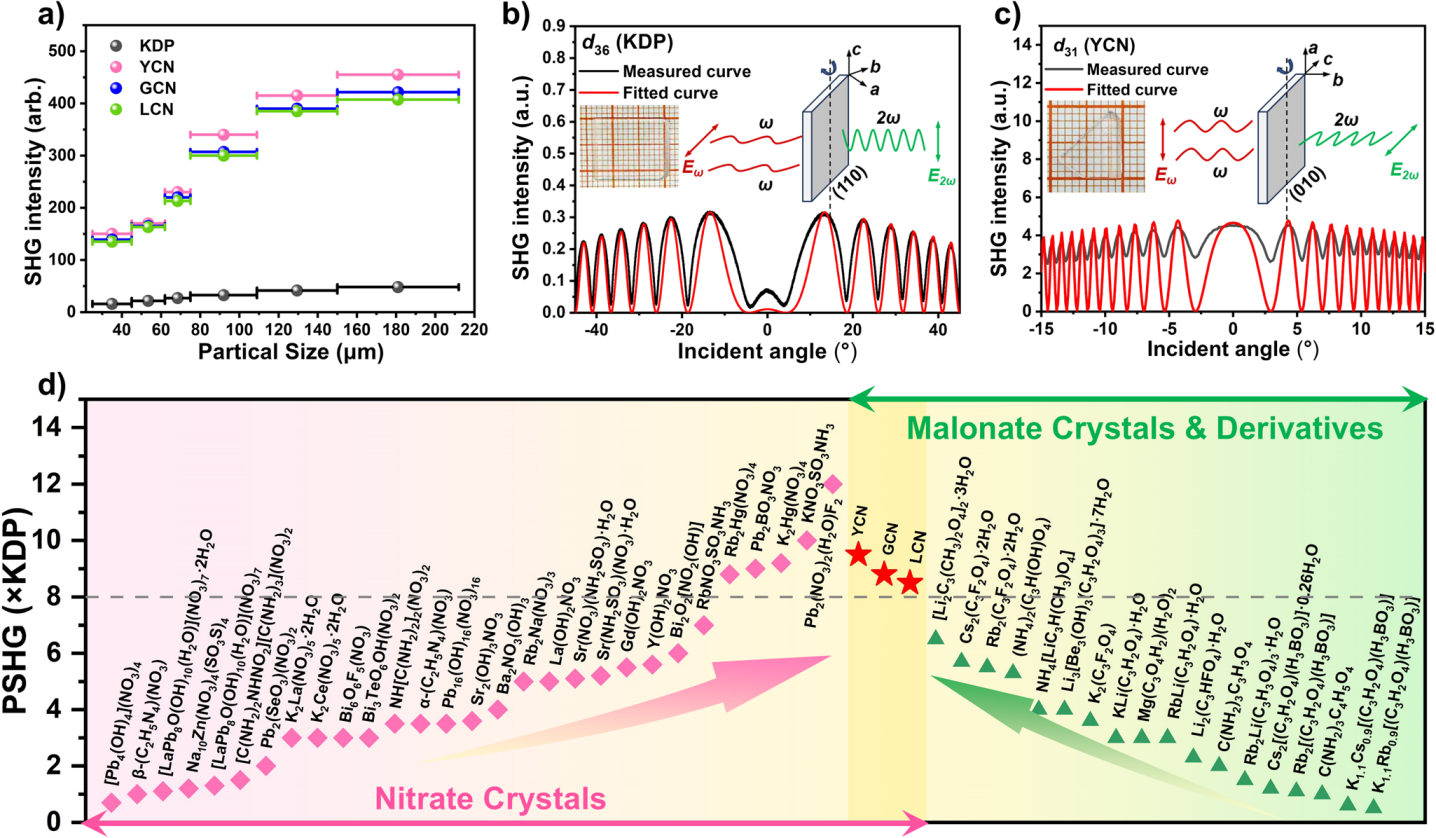
(a) Powder SHG (PSHG) data for YCN, GCN, and LCN at 1064 nm; (b, c) the Maker fringes of *d*_36_ (KDP) and *d*_31_ (YCN); (d) statistical comparison of PSHG for nitrates and malonates with their derivatives.

The powder SHG experiments were conducted following the Kurtz‐Perry method, employing a Q‐switched Nd: YAG laser (wavelength: 1064 nm), with polycrystalline KDP crystals as a reference. Figure 5a revealed that YCN, GCN, and LCN all exhibit phase matching behaviors as the intensities increase with increasing particle sizes [50]. YCN, GCN, and LCN exhibited 9.5×, 8.8×, and 8.5× KDP, respectively. Since YCN, GCN, and LCN all crystallize in the space group *Pmn*2_1_, three non‐zero independent SHG coefficients (*d*_31_ = *d*_15_, *d*_32_ = *d*_24_, *d*_33_) are present under the restriction of Kleinman symmetry (Figure S12). Relative calculations reveal *d*_31_ of YCN, GCN, and LCN are the largest SHG coefficient to be 4.48, 4.05, and 3.77 pm/V, respectively. Through Equation S2, the calculated *d_eff_* values of YCN, GCN, and LCN under 1064 nm irradiation are 2.80, 2.51, and 2.42 pm/V, respectively, corresponding to 8.5×, 7.6×, and 7.3× KDP ($d_{eff}^{KDP}$= 0.33 pm/V) [51, 52]. These calculated values are consistent with the experimental observations. Since the *d*_31_ coefficient dominates the SHG effect, the *d*_31_ of YCN were further measured using Maker fringes technology (with KDP crystals as a reference; Figure 5b,c) [34, 53, 54]. Then, the data were fitted based on Maker fringe theory, yielding a *d*_31_ value of 4.22 pm/V, proving the accuracy of our calculated values.

**FIGURE 6:**
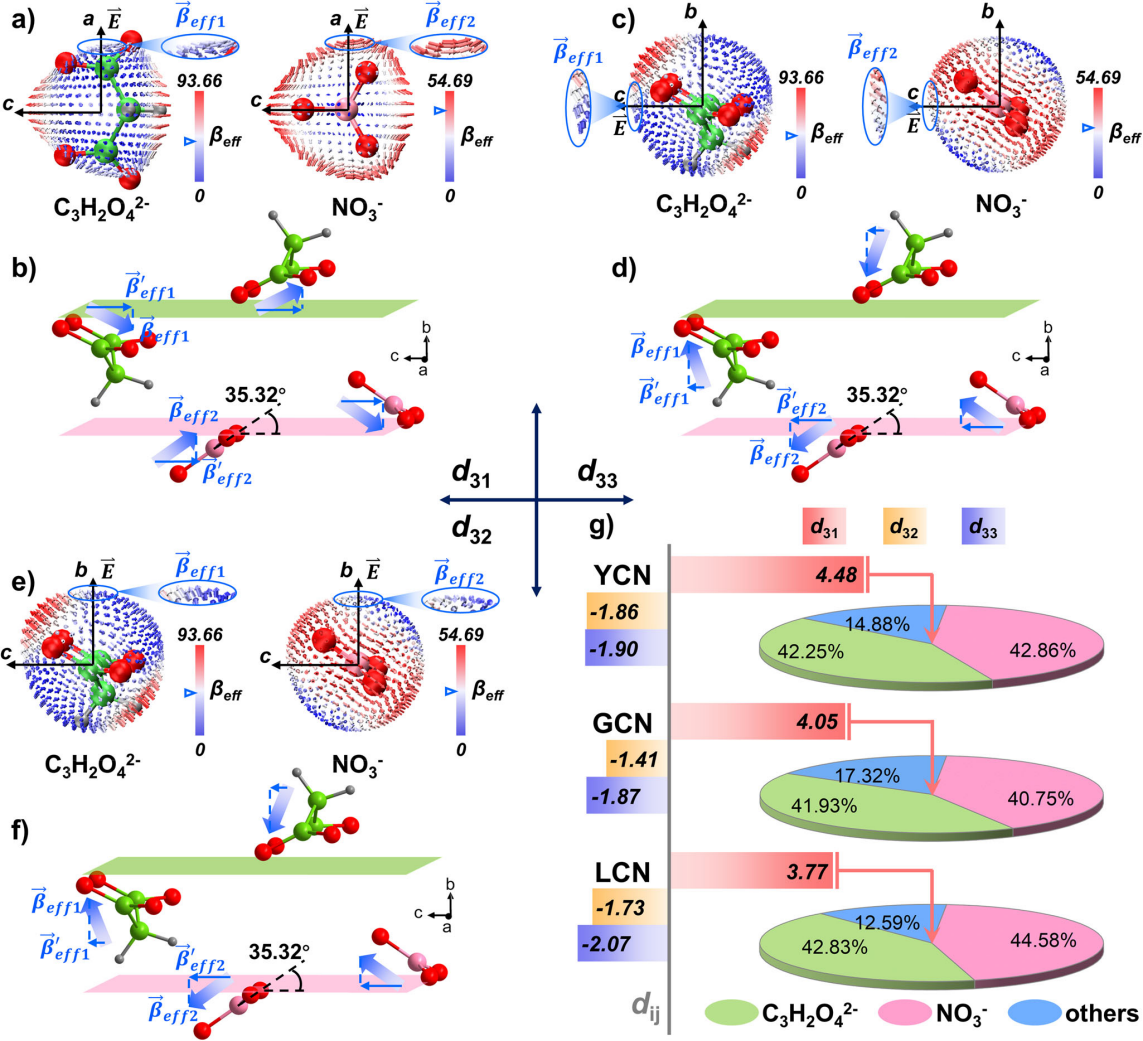
(a, c, e) The unit sphere representation of static hyperpolarizability tensors for C_3_H_2_O_4_^2−^ and NO_3_^−^ (arrows represent the unit sphere vectors of the hyperpolarizability tensors, including magnitude and direction: redder, longer arrows signify higher hyperpolarizability, while bluer, shorter ones indicate smaller hyperpolarizability. The small arrow at the intersection of the unit sphere and the polarization direction of $\vec{E}$ denotes the relative ${\vec{\beta }}_{eff}$ of the unit); (b, d, f) the orientations of ${\vec{\beta }}_{eff}$ tensors and the components of ${\vec{\beta }}_{eff}$ along the *c*‐axis (i.e., ${{\vec{\beta }}^{\prime }}_{eff}$); g) the calculated SHG coefficients and atom‐cutting analysis of *d*_31_ for YCN, GCN, and LCN.

The SHG density maps for *d*_31_ visually reveal that the C_3_H_2_O_4_^2−^ and NO_3_^−^ groups dominant the SHG responses of YCN, GCN, and LCN (Figure S13), consisting with the classical anionic theory. According to this theory, the hyperpolarizability (*β*), arrangement, and density of anionic groups collectively determine the macroscopic SHG response. Therefore, the significant hyperpolarizability of units (*β*_max_ = 93.66 a.u. for C_3_H_2_O_4_^2−^ and *β*_max_ = 54.69 a.u. for NO_3_^−^), the relatively neat arrangement along the *c*‐axis, and considerable group density (*ρ* = 4.037, 3.945, and 3.999 × 10^−3^ Å^−3^ for either C_3_H_2_O_4_^2−^ and NO_3_^−^ units in YCN, GCN, and LCN, respectively) collaboratively contribute to the large SHG effect for YCN, GCN, and LCN. To gain deeper insights of the relationship between units and SHG performance, the unit sphere representations were computed to intuitively depict the effective hyperpolarizability (${\vec{\beta }}_{eff}$) for relative SHG coefficients (Figure 6) [55]. The unit sphere representation maps of C_3_H_2_O_4_^2−^ and NO_3_^−^ units reveal that larger hyperpolarizability tensors distribute in the plane of groups. For the SHG coefficient *d*_31_, when the fundamental frequency wave is polarized along the *a*‐axis (i.e., $\vec{E}$ is along *a*‐axis), the corresponding intersecting arrows (i.e., ${\vec{\beta }}_{eff}$) of C_3_H_2_O_4_^2−^ and NO_3_^−^ are both nearly aligned along the ‐*c*‐axis with comparable magnitude around 40 a.u., as indicated by the color table (Figure 6a). Then, along the crystal orientation of frequency‐doubled wave (the *c*‐axis), the components of ${\vec{\beta }}_{eff}$ along the *c*‐axis (i.e., ${{\vec{\beta }}^{\prime }}_{eff}$) for both anionic groups superimpose, yielding the maximum *d*_31_ coefficient (Figure 6b). Additionally, the similar magnitude and arrangement of ${\vec{\beta }}_{eff}$ for the C_3_H_2_O_4_^2−^ and NO_3_^−^ groups should contribute nearly equally to *d*_31_. Atom‐cutting analysis revealed that the calculated SHG‐contribution percentages of C_3_H_2_O_4_^2−^ and NO_3_^−^ are nearly identical: 42.25% and 42.86% in YCN, 41.93% and 40.75% in LCN, and 42.83% and 44.58% in LCN, respectively (Figure 6g; Table S7). However, when $\vec{E}$ is along *c*‐axis and *b*‐axis, the relative arrows outside the group plane are smaller or deviate significantly from *c*‐axis, which can help explain why the *d*_33_ and *d*_32_ are small (Figure 6c–g).

The dipole moment describes the asymmetry of static charge distribution, characterizing the polarity of a group. The hyperpolarizability describes the deformability of the electron cloud in an electric field, characterizing the group's nonlinear response to an optical electric field. Specifically, the consistent superposition of dipole moments facilitates non‐centrosymmetric structures, while that of hyperpolarizabilities enhances SHG efficiency. Importantly, no simple positive correlation exists between the dipole moment and hyperpolarizability. Therefore, although the C_3_H_2_O_4_^2−^ and NO_3_^−^ groups have opposite dipoles, their combination contributes positively to the SHG effect. The YCN, GCN, and LCN crystals exhibited SHG efficiencies of 9.5, 8.8, and 8.5 times that of KDP, respectively. These values represent some of the largest SHG effects for NLO nitrates and the highest SHG effects for NLO malonates to date (Figure 5d; Tables S8 and S9). Among nitrates with SHG responses greater than 8.0× KDP (excluding this study), only five compounds have shown higher values: Rb_2_Hg(NO_3_)_4_ (8.8×) [40], K_2_Hg(NO_3_)_4_ (9.2×) [40], Pb_2_BO_3_NO_3_ (9.0×) [56], KNO_3_SO_3_NH_3_ (10.0×) [46], Pb_2_(NO_3_)_2_(H_2_O)F_2_ (12.0×) [57]. However, the size advantages and non‐toxic nature of YCN, GCN, and LCN make them more practical for real‐world applications. For malonates and their derivatives, YCN, GCN, and LCN have surpassed the previously established SHG response limit of 8.0× KDP, such as [Li_2_C_3_(CH_3_)_2_O_4_]_2_·3H_2_O (6.5×), Cs_2_(C_3_F_2_O_4_)·2H_2_O (5.7×), and Rb_2_(C_3_F_2_O_4_)·2H_2_O (5.5×), thus enhancing overall conversion efficiency [58]. In conclusion, guided by the *RPIO* strategy and the “A‐B‐A” linkage model, the synergistic integration of strongly polar C_3_H_2_O_4_^2−^ units with weakly polar NO_3_^−^ units not only enables the controllability of layer stacking but also significantly enhances SHG responses.

## 3 Conclusion

In summary, we present a novel “*Reverse Weak Polarity‐Induced Ordered Layer Control*” (RPIO) strategy to achieve consistent alignment of polar layers via a “layer→linker→layer” polarity transfer mechanism. The strategy is operationalized through the design of an “A‐B‐A” linkage model, and three isostructural crystals—RE(C_3_H_2_O_4_)NO_3_·4H_2_O (RE = Y, Gd, Lu)—were successfully synthesized as the proof. In these compounds, strongly polar [RE(C_3_H_2_O_4_)(H_2_O)_4_]+ ∞ A‐layers exhibit coherently alignment induced by weakly polar, reverse‐oriented NO_3_^−^ B‐linkers, with directional hydrogen bonds mediating interlayer coupling. This interlayer engineering promotes uniform arrangement of functional groups within and across layers, resulting in exceptional SHG responses (8.5–9.5× KDP) of RE(C_3_H_2_O_4_)NO_3_·4H_2_O (RE = Y, Gd, Lu). Additionally, they feature wide bandgaps (∼4.13 eV), birefringence (∼0.135 @589.3 nm), and favorable growth habits, highlighting their promise for UV NLO applications. More broadly, this work demonstrates that systematic regulation of linkers and interlayer coupling provides a predictive and tunable route to control layer stacking, thereby enhancing the macroscopic SHG in layered UV NLO materials.

## 4 Experimental Section/Methods

Details of the experiments and methods are provided in the Supporting Information.

## Funding

This work was supported by the National Natural Science Foundation of China (22471271, 52302008, 22505261), the Natural Science Foundation of Fujian Province (2023J02026, 2024J08102, 2025J01244), Fuzhou Science and Technology Program (2025‐P‐001), the Self‐deployed Project of State Key Laboratory of Functional Crystals and Devices (GNJT‐2025‐QN03, ZRQN2501F), and the Youth Innovation Promotion Association CAS (Y2023082).

## Conflicts of Interest

None of the authors have a conflict of interest to disclose.

## Supporting information




**Supporting File**: advs75642‐sup‐0001‐SuppMat.docx.

**Supporting data**: advs75642‐sup‐0002‐Data.zip.

## Data Availability

The data that supports the findings of this study are available in the supplementary material of this article. [CCDC 2526475–2526477 contains the supplementary crystallographic data for this paper. These data can be obtained free of charge from The Cambridge Crystallographic Data Centre via www.ccdc.cam.ac.uk/data_request/cif.]
