# Supplementary material for: Reverse Weak Polarity‐Induced Ordered Layer Control for Enhanced Second‐Harmonic Generation in Ultraviolet Nonlinear Optical Crystals
Source: Adv Sci (Weinh). 2026 May 11;13(43):e75642. doi: 10.1002/advs.75642 (PMC13335983; doi:10.1002/advs.75642)
Supplement: Supplementary file 1 — Supporting File: advs75642‐sup‐0001‐SuppMat.docx. [file ADVS-13-e75642-s002.docx]

Supporting Information

Reverse Weak Polarity-Induced Ordered Layer Control for Enhanced Second-Harmonic Generation in Ultraviolet Nonlinear Optical Crystals

Lingli Wu, Chensheng Lin, Bing-Xuan Li, Huixin Fan, Lingfei Lv, Shunda Yang, Tao Yan, and Min Luo*

**Experimental and Computational Methods**

**1 Reagents**

C_3_H_4_O_4_ (Adamas, 99.0%), Y(NO_3_)_3_·6H_2_O (Adamas, 99.9%), Gd(NO_3_)_3_·6H_2_O (Adamas, 99.95%), Lu(NO_3_)_3_·6H_2_O (Adamas, 99.95%), Li_2_CO_3_ (Adamas, 99%+).

**2 Synthesis**

**Pure phase**

RE(C_3_H_2_O_4_)NO_3_·4H_2_O (RE= Y, Gd, Lu): Dissolved 0.010 mol C_3_H_4_O_4_, 0.005 mol RE(NO_3_)_3_·6H_2_O, and 0.100 g Li_2_CO_3_ in 15 mL deionized water, and then allow the solution to slowly evaporate in 35 ℃ oven to crystallize. Without the addition of Li_2_CO_3_ or LiOH, crystallization cannot occur.

**Single crystal growth of** Y(C_3_H_2_O_4_)NO_3_·4H_2_O

Dissolved 31.218 g of C_3_H_4_O_4_, 67.704 g of Y(NO_3_)_3_·6H_2_O, and 0.500 g Li_2_CO_3_ in 100 mL deionized water, and then allow the solution to slowly evaporate in 35 ℃ oven to crystallize.

**3 Single-Crystal Structure Determination**

Single-crystal X-ray diffraction (SC-XRD) data were collected on a Rigaku Mercury CCD diffractometer equipped with graphite-monochromatic Cu-Kα radiation (λ = 1.5406 Å). The crystal structures of RE(C_3_H_2_O_4_)NO_3_·4H_2_O (RE= Y, Gd, Lu) were solved by using the ShelXT, and then refined by the ShelXL on OLEX2 package.^[1]^ The program PLATON was used to check for the structure. Crystallographic data and structure refinements of RE(C_3_H_2_O_4_)NO_3_·4H_2_O (RE= Y, Gd, Lu) were given in the Table S1. Atomic coordinates and equivalent isotropic displacement parameters, hydrogen coordinates and isotropic displacement parameters, anisotropic displacement parameters, and Bond lengths and angles for RE(C_3_H_2_O_4_)NO_3_·4H_2_O (RE= Y, Gd, Lu) were shown in Table S2-S5.

**4 Powder X-ray diffraction (PXRD) and Rietveld refinement of PXRD**

The PXRD data of I and II were measured on a Miniflex600 powder X-ray diffractometer with Cu Kα radiation (λ = 1.5406 Å) in the 2θ range from 10° to 80°.

**5 Energy-dispersive X-ray Spectroscopy Analysis**

Microprobe elemental analyses were recorded on a field emission scanning electron microscope (FESEM, SU-8010) with an energy dispersive X-ray spectroscope (EDS).

**6 Thermal analysis**

Thermogravimetric Analyses (TGA) were performed on a Netzsch STA449F3 simultaneous analyzer under flowing N_2_ at a rate of 10 °C·min^-1^.

**7 The UV/Vis diffuse-reflectance Spectrums UV-Vis-NIR transmittance Spectrums**

The UV diffuse-reflectance and transmittance Spectrums of RE(C_3_H_2_O_4_)NO_3_·4H_2_O (RE= Y, Gd, Lu) were measured using the McPherson VUV as 2000 spectrophotometer at room temperature.

**8 Birefringence measurement and optic axial angle (2*V*) value calculation**

Refractive indices are measured through the immersion technique using an FGE-002A Gem refractometer with 589.3 nm light. 2*V* value of biaxial crystal with *Ng*> *Nm*> *Np*:

$sin\theta=\frac{Ng}{Nm}\sqrt{({Nm}^{2}-{Np}^{2})/({Ng}^{2}-{Np}^{2})}$ (**Equation S1**)

Where *θ* is the angle between either optic axis and the axis of *Ng*.

As for the negative optical crystals (*Ng*- *Nm*< *Nm*- *Np*), 2*V*= 2🞩(π/2- *θ*).

**9 Second-Harmonic Generation measurement**

Polycrystalline second-harmonic generation (SHG) signals for RE(C_3_H_2_O_4_)NO_3_·4H_2_O (RE= Y, Gd, Lu) were measured by the Kurtz-Perry method on 1064nm solid-state laser with KDP as reference samples.^[2]^ The crystals were ground and sieved into 25-45, 45-62, 62-75, 75-109, 109-150, and 150-212 μm.

**10 Effective SHG response *d*_eff_**

The *d*_eff_ value is estimated from the Equation S2 derived by Kurtz-Perry and Cyvin et al.^[2,3]^

$d_{kp}^{2}=\frac{5}{7}(d_{ijk})^{2}+\frac{19}{105}\sum_{i} (d_{iii})^{2}+\frac{13}{105}\sum_{i\neq j} \left( d_{iii}d_{ijj} \right)+\frac{44}{105}\sum_{i\neq j} (d_{iij})^{2}+\frac{13}{105}\sum_{ijk,cyclic} \left( d_{iij}d_{jkk} \right),$ (**Equation S2**)

The SHG tensors *d*_ijk_ are calculated through the formula proposed by Sipe et al, and the subscript ijk can be any cyclic combination of the indices, i.e., 123, 312, and 231.

**11 Maker fringe measurements**

Maker fringe measurements were carried out on a (010)-oriented YCN wafer to determine the nonlinear optical coefficients *d*_31_. The experimental data were subsequently analyzed based on Maker fringe theory. The detected SHG power *P*_2_*_ω_* is given by Equation S3:

$P_{2\omega}\left( \theta\right)=\frac{512\pi^{2}}{c\omega^{2}}d^{2}P_{\omega}^{2}f(n, \theta){sin}^{2}\Psi$ (**Equation S3**)

Here, $f(n, \theta)$ is defined as:

$f\left( n, \theta\right)=\frac{1}{{(n}_{\omega}^{2}-n_{2\omega}^{2})^{2}}t_{\omega}^{4}T_{2\omega}P^{2}(\theta)R(\theta)\beta(\theta)$ (**Equation S4**)

In the above expressions, *c* is the speed of light in vacuum, *ω* denotes the beam radius, and *n_ω_* and *n_2ω_* are the refractive indices at the fundamental and second-harmonic wavelengths, respectively. *d* represents the NLO coefficient, and *P_ω_* is the the fundamental beam power. The parameter Ψ is expressed as:

$\Psi=\frac{2\pi L(n_{\omega}cos\theta_{\omega}-n_{2\omega}cos\theta_{2\omega})}{\lambda_{\omega}}$ (**Equation S5**)

The term sin^2^Ψ governs the minima in the interference pattern. Furthermore, *t_ω_* and *t_2ω_* are the transmission coefficients for the fundamental and harmonic waves, *p(θ)* is the projection factor, *R(θ)* corrects for multiple reflections at the interfaces, and *β(θ)* corrects for variations in beam size. The function $f(n, \theta)$ describes the envelope of the Maker fringe pattern.

By fitting the theoretical calculations to the experimentally measured Maker fringes, a constant parameter *C* can be extracted:

$C=\frac{512\pi d^{2}P_{\omega}^{2}}{c\omega^{2}}$ (**Equation S6**)

To determine the magnitude of the YCN's NLO coefficients, a relative comparison is made with the *d*_36_ coefficient of a KDP reference crystal. The NLO coefficient for the sample is then obtained using the following relation:

$d_{sample}=\sqrt{\frac{C_{sample}}{C_{KDP}}}d_{36}(KDP)$ (**Equation S7**)

**12 First-principles calculations**

The CASTEP package, which utilizes density functional theory (DFT),^[4]^ was used to calculate the band structures of RE(C_3_H_2_O_4_)NO_3_·4H_2_O (RE= Y, Gd, Lu). The exchange-correlation energy^[4]^ was treated by the generalized gradient approximation (GGA) with Perdew-Burke-Ernzerhof (PBE)^[5]^ functional. The valence electrons consider were: H 1*s*^1^; C 2*s*^2^2*p*^2^; N 2*s*^2^2*p*^3^; O 2*s*^2^2*p*^4^; Y 4*d*^1^5*s*^2^; Gd 4*f*^7^5*d*^1^6*s*^2^; Lu 4*f*^14^5*d*^1^6*s*^2^. For RE(C_3_H_2_O_4_)NO_3_·4H_2_O (RE= Y, Gd, Lu), the energy cutoff for the calculation were set to 750, 820, and 900 eV, respectively. The Monkhorst-Pack grid size used were all 2×2×2. The GGA functional was then used to calculate the band gaps for RE(C_3_H_2_O_4_)NO_3_·4H_2_O (RE= Y, Gd, Lu). In order to match the calculated GGA band gaps with the measured values in the optical-property calculations, scissor operators^[6,7]^ of 0.379 eV, 0.453 eV and 0.457 eV were applied to shift all the conduction bands. Based on the scissor-corrected electron band structure, the imaginary part of the dielectric function was calculated. The real part was obtained by the Kramers-Kronig transform, and then the refractive index was determined. The SHG coefficients *d_ijk_* were obtained by the formula originally proposed by Sipe *et al*^[8]^ and developed by Cheng *et al*.^[9]^ The SHG densities of *d*_31_ were calculated out by the band-resolved method.^[10]^ The SHG-contribution percentage of fragments of *d*_31_ were calculated through the Bader charge analysis.

The interaction energy decomposition between NO_3_^-^ and H_2_O fragments was calculated based on symmetry-adapted perturbation theory (SAPT) at the second-order perturbation SAPT2+/ aug-cc-pVDZ level implemented in sobEDAw-cal program.^[11,12]^ The dipolar and the hyperpolarizability tensor were calculated through the B3LYP/6-31G**. Vector Representation of the polarizability tensor were calculated by B3LYP/6-311^++^G** method, and parsed through Multiwfn.^[13,14]^

**Table S1.** Crystal data and structure refinements for RE(C_3_H_2_O_4_)NO_3_·4H_2_O (RE=Y, Gd, Lu).

| Formula | Y(C_3_H_2_O_4_)NO_3_·4H_2_O | Gd(C_3_H_2_O_4_)NO_3_·4H_2_O | Lu(C_3_H_2_O_4_)NO_3_·4H_2_O |
| --- | --- | --- | --- |
| Formula weight (g/mol) | 325.03 | 393.37 | 205.54 |
| Temperature/K | 100(2) | 99.99(10) | 294(2) |
| Crystal system | Orthorhombic | Orthorhombic | Orthorhombic |
| space group | *Pmn*2_1_ | *Pmn*2_1_ | *Pmn*2_1_ |
| *a*/Å | 8.0577(3) | 8.0875(3) | 8.0469(2) |
| *b*/Å | 6.6990(2) | 6.73074(18) | 6.83633(17) |
| *c*/Å | 9.1976(3) | 9.3125(3) | 9.0923(2) |
| *α*/° | 90 | 90 | 90 |
| *β*/° | 90 | 90 | 90 |
| *γ*/° | 90 | 90 | 90 |
| *V*/Å^3^ | 496.47(3) | 506.92(3) | 500.18(2) |
| *Z* | 2 | 2 | 4 |
| *ρ_calc_* (g/cm^3^) | 2.174 | 2.577 | 2.730 |
| *μ*/mm^-1^ | 5.929 | 6.596 | 9.923 |
| *F*(000) | 324 | 374 | 388 |
| *θ*/° | 3.041 - 31.355 | 3.026 - 31.457 | 2.980 - 30.100 |
| Limiting indices | -11<=h<=11 | -11<=h<=7 | -11<=h<=11 |
|  | -9<=k<=9 | -8<=k<=9 | -9<=k<=9 |
|  | -12<=l<=13 | -13<=l<=12 | -12<=l<=12 |
| Reflections collected/unique | 11739 / 1616 | 5812 / 1537 | 10997 / 1387 |
| *R_int_* | 0.0932 | 0.0499 | 0.0784 |
| GOF on *F^2^* | 1.130 | 1.089 | 1.090 |
| *R_1_/wR_2_* [I>2σ(I)] | 0.0308 / 0.0711 | 0.0269 / 0.0587 | 0.0303 / 0.0852 |
| *R_1_/wR_2_* (all data) | 0.0323 / 0.0716 | 0.0275 / 0.0592 | 0.0307 / 0.0855 |
| Largest difference peak and hole (e. Å^-3^) | 0.847 and -1.559 | 1.191 and -1.227 | 1.064 and -1.243 |
| Absolute structure parameter | -0.044(6) | 0.002(19) | -0.072(13) |

*R_1_(F) =Σ||F_o_| - |F_c_||/Σ|F_o_|; wR_2_ (F_o_^2^) = [Σw(F_o_^2^ – F_c_^2^)^2^/Σw(F_o_^2^)^2^]^1/2^*

**Table S2.** Atomic coordinates (x10^4^) and equivalent isotropic displacement parameters (Å^2^x10^3^) for RE(C_3_H_2_O_4_)NO_3_·4H_2_O (RE=Y, Gd, Lu).

| Compound | Atom | x | y | z | U(eq) |
| --- | --- | --- | --- | --- | --- |
| Y(C_3_H_2_O_4_)NO_3_·4H_2_O | Y(1) | -5000 | 172(1) | -6102(1) | 6(1) |
|  | O(1) | -5000 | 657(7) | -3540(4) | 11(1) |
|  | O(2) | -3326(4) | 3033(5) | -5682(4) | 23(1) |
|  | O(3) | -3255(4) | 524(5) | -8116(3) | 11(1) |
|  | O(4) | -2595(4) | -1329(5) | -5349(3) | 25(1) |
|  | O(5) | -5000 | -3225(6) | -6990(5) | 18(1) |
|  | O(6) | -1337(6) | -5196(6) | -8204(6) | 43(1) |
|  | O(7) | 0 | -6813(6) | -6542(4) | 12(1) |
|  | C(1) | -1565(4) | -1382(5) | -4328(4) | 11(1) |
|  | C(2) | 0 | -2573(8) | -4612(6) | 9(1) |
|  | N(1) | 0 | -5731(7) | -7677(5) | 12(1) |
| Gd(C_3_H_2_O_4_)NO_3_·4H_2_O | Gd(1) | 10000 | 167(1) | 4185(1) | 6(1) |
|  | O(1) | 10000 | -3261(9) | 3295(8) | 21(2) |
|  | O(2) | 8298(6) | 3072(8) | 4611(5) | 30(1) |
|  | O(3) | 10000 | 673(10) | 6765(7) | 11(1) |
|  | O(4) | 7571(7) | -1338(9) | 4946(6) | 32(1) |
|  | O(5) | 6764(6) | -561(7) | 7158(5) | 13(1) |
|  | O(6) | 8685(11) | 5233(10) | 7038(10) | 60(3) |
|  | O(7) | 10000 | 6738(9) | 8723(6) | 13(1) |
|  | C(1) | 6559(8) | -1396(9) | 5960(7) | 13(1) |
|  | C(2) | 5000 | -2612(12) | 5701(10) | 10(2) |
|  | N(1) | 10000 | 5726(12) | 7569(9) | 16(2) |
| Lu(C_3_H_2_O_4_)NO_3_·4H_2_O | Lu(1) | 5000 | 200(1) | 5677(1) | 19(1) |
|  | O(1) | 3262(9) | 541(11) | 3663(8) | 18(1) |
|  | O(2) | 5000 | -3101(15) | 4766(12) | 26(2) |
|  | O(3) | 7405(8) | -1305(11) | 6424(7) | 21(1) |
|  | O(4) | 5000 | 642(17) | 8242(11) | 19(2) |
|  | O(5) | 6655(9) | 2964(12) | 6090(8) | 26(2) |
|  | O(6) | 10000 | 3208(14) | 5222(9) | 22(2) |
|  | O(7) | 8637(16) | 4846(12) | 3603(14) | 43(3) |
|  | C(1) | 3437(9) | 1344(12) | 2440(9) | 13(2) |
|  | C(2) | 5000 | 2503(19) | 2148(15) | 16(2) |
|  | N(1) | 10000 | 4300(19) | 4108(13) | 21(2) |

**Table S3.** Hydrogen coordinates (x10^4^) and isotropic displacement parameters (Å^2^x10^3^) for RE(C_3_H_2_O_4_)NO_3_·4H_2_O (RE=Y, Gd, Lu).

| Compound | Atom | x | y | z | U(eq) |
| --- | --- | --- | --- | --- | --- |
| Y(C_3_H_2_O_4_)NO_3_·4H_2_O | H(1) | -4053 | 136 | -2994 | 17 |
|  | H(2A) | -3540 | 3580 | -4797 | 35 |
|  | H(2B) | -2520(100) | 2920(160) | -5770(110) | 90(30) |
|  | H(2C) | 0 | -3029 | -5636 | 11 |
|  | H(2D) | 0 | -3772 | -3983 | 11 |
| Gd(C_3_H_2_O_4_)NO_3_·4H_2_O | H(1) | 9026 | -3834 | 3463 | 31 |
|  | H(2A) | 8497 | 3592 | 5487 | 44 |
|  | H(2B) | 7222 | 2730 | 4661 | 44 |
|  | H(2C) | 5000 | -3106 | 4699 | 12 |
|  | H(2D) | 5000 | -3778 | 6348 | 12 |
| Lu(C_3_H_2_O_4_)NO_3_·4H_2_O | H(2A) | 4162 | -3808 | 4562 | 39 |
|  | H(2B) | 5833 | -3824 | 4580 | 39 |
|  | H(2C) | 5000 | 2932 | 1131 | 19 |
|  | H(2D) | 5000 | 3660 | 2766 | 19 |
|  | H(4A) | 4113 | 41 | 8710 | 29 |
|  | H(4B) | 5884 | 38 | 8714 | 29 |
|  | H(5A) | 6480(120) | 3740(110) | 6770(90) | 38 |
|  | H(5B) | 7250(120) | 3420(110) | 5450(70) | 38 |

**Table S4.** Anisotropic displacement parameters (Å^2^x10^3^) for RE(C_3_H_2_O_4_)NO_3_·4H_2_O (RE=Y, Gd, Lu). The anisotropic displacement factor exponent takes the form: -2π^2^[h^2^a*^2^U_11_+…+2hka*b*U_12_].

| Compound | Atom | U11 | U22 | U33 | U23 | U13 | U12 |
| --- | --- | --- | --- | --- | --- | --- | --- |
| Y(C_3_H_2_O_4_)NO_3_·4H_2_O | Y(1) | 5(1) | 7(1) | 6(1) | 0(1) | 0 | 0 |
|  | O(1) | 10(2) | 16(2) | 9(2) | 1(2) | 0 | 0 |
|  | O(2) | 14(2) | 29(2) | 25(2) | -18(1) | 11(1) | -11(1) |
|  | O(3) | 10(1) | 13(1) | 9(1) | 2(1) | 2(1) | 3(1) |
|  | O(4) | 21(2) | 38(2) | 17(2) | -14(1) | -10(1) | 20(1) |
|  | O(5) | 24(2) | 11(2) | 20(2) | -2(2) | 0 | 0 |
|  | O(6) | 29(2) | 50(3) | 49(3) | 37(2) | -30(2) | -23(2) |
|  | O(7) | 13(2) | 13(2) | 11(2) | 3(1) | 0 | 0 |
|  | C(1) | 11(2) | 11(2) | 12(2) | -2(1) | -2(1) | 3(1) |
|  | C(2) | 8(2) | 8(2) | 13(3) | 0(2) | 0 | 0 |
|  | N(1) | 17(2) | 8(2) | 12(2) | 0(2) | 0 | 0 |
| Gd(C_3_H_2_O_4_)NO_3_·4H_2_O | Gd(1) | 8(1) | 7(1) | 5(1) | 0(1) | 0 | 0 |
|  | O(1) | 37(4) | 10(3) | 16(3) | -3(3) | 0 | 0 |
|  | O(2) | 22(3) | 36(3) | 31(3) | -23(2) | -15(2) | 16(2) |
|  | O(3) | 13(3) | 13(3) | 8(3) | -2(3) | 0 | 0 |
|  | O(4) | 29(3) | 49(4) | 19(3) | -19(2) | 16(2) | -28(3) |
|  | O(5) | 14(2) | 15(2) | 10(2) | -4(2) | 4(2) | -5(2) |
|  | O(6) | 47(5) | 66(5) | 67(6) | -56(4) | -44(5) | 40(4) |
|  | O(7) | 15(3) | 14(3) | 10(3) | -2(2) | 0 | 0 |
|  | C(1) | 16(3) | 13(3) | 12(3) | -4(2) | 0(2) | -6(2) |
|  | C(2) | 8(4) | 8(4) | 13(4) | -1(3) | 0 | 0 |
|  | N(1) | 29(5) | 4(3) | 14(4) | 0(3) | 0 | 0 |
| Lu(C_3_H_2_O_4_)NO_3_·4H_2_O | Lu(1) | 16(1) | 29(1) | 13(1) | 0(1) | 0 | 0 |
|  | O(1) | 12(3) | 28(3) | 12(3) | 5(3) | 1(2) | 0(3) |
|  | O(2) | 20(4) | 22(5) | 36(5) | -10(4) | 0 | 0 |
|  | O(3) | 17(3) | 33(4) | 14(3) | -6(3) | -11(2) | 11(3) |
|  | O(4) | 12(4) | 31(5) | 14(4) | 0(4) | 0 | 0 |
|  | O(5) | 23(3) | 30(4) | 24(4) | -9(2) | 12(2) | -10(3) |
|  | O(6) | 23(4) | 23(5) | 19(4) | 10(3) | 0 | 0 |
|  | O(7) | 37(6) | 47(6) | 46(7) | 26(4) | -20(5) | -10(4) |
|  | C(1) | 13(3) | 16(4) | 10(3) | -1(3) | 1(3) | 1(3) |
|  | C(2) | 4(4) | 21(6) | 22(6) | 7(5) | 0 | 0 |
|  | N(1) | 25(6) | 19(5) | 18(5) | 2(5) | 0 | 0 |

**Table S5.** Bond lengths [Å] and angles [°] for RE(C_3_H_2_O_4_)NO_3_·4H_2_O (RE=Y, Gd, Lu).

| Y(C_3_H_2_O_4_)NO_3_·4H_2_O | | | | | |
| --- | --- | --- | --- | --- | --- |
| Y(1)-O(4) | 2.291(3) | O(4)-Y(1)-O(3) | 76.99(11) | O(2)#1-Y(1)-O(5) | 144.52(9) |
| Y(1)-O(4)#1 | 2.291(3) | O(4)#1-Y(1)-O(3) | 142.45(11) | O(1)-Y(1)-O(5) | 117.61(15) |
| Y(1)-O(3)#1 | 2.338(3) | O(3)#1-Y(1)-O(3) | 73.97(14) | Y(1)-O(5)-H(5) | 114.2 |
| Y(1)-O(3) | 2.338(3) | O(4)-Y(1)-O(2) | 79.90(14) | C(1)#2-O(3)-Y(1) | 132.6(2) |
| Y(1)-O(2) | 2.375(3) | O(4)#1-Y(1)-O(2) | 141.78(12) | Y(1)-O(2)-H(2A) | 111.3 |
| Y(1)-O(2)#1 | 2.376(3) | O(3)#1-Y(1)-O(2) | 112.89(13) | Y(1)-O(2)-H(2B) | 117(9) |
| Y(1)-O(1) | 2.379(4) | O(3)-Y(1)-O(2) | 72.88(11) | H(2A)-O(2)-H(2B) | 109.9 |
| Y(1)-O(5) | 2.418(4) | O(4)-Y(1)-O(2)#1 | 141.78(12) | C(1)-O(4)-Y(1) | 143.0(3) |
| O(5)-H(5) | 1.0302 | O(4)#1-Y(1)-O(2)#1 | 79.90(14) | O(6)-N(1)-O(6)#3 | 121.5(6) |
| O(3)-C(1)#2 | 1.263(5) | O(3)#1-Y(1)-O(2)#1 | 72.88(11) | O(6)-N(1)-O(7) | 119.2(3) |
| O(2)-H(2A) | 0.9092 | O(3)-Y(1)-O(2)#1 | 112.89(13) | O(6)#3-N(1)-O(7) | 119.2(3) |
| O(2)-H(2B) | 0.66(8) | O(2)-Y(1)-O(2)#1 | 69.21(18) | Y(1)-O(1)-H(1) | 117.4 |
| O(4)-C(1) | 1.254(5) | O(4)-Y(1)-O(1) | 76.14(9) | O(4)-C(1)-O(3)#4 | 124.9(3) |
| O(7)-N(1) | 1.271(6) | O(4)#1-Y(1)-O(1) | 76.14(9) | O(4)-C(1)-C(2) | 115.9(3) |
| N(1)-O(6) | 1.235(5) | O(3)#1-Y(1)-O(1) | 140.46(8) | O(3)#4-C(1)-C(2) | 119.2(3) |
| N(1)-O(6)#3 | 1.235(5) | O(3)-Y(1)-O(1) | 140.46(8) | C(1)-C(2)-C(1)#3 | 112.7(4) |
| O(1)-H(1) | 0.9782 | O(2)-Y(1)-O(1) | 74.27(12) | C(1)-C(2)-H(2C) | 109.1 |
| C(1)-C(2) | 1.515(5) | O(2)#1-Y(1)-O(1) | 74.27(12) | C(1)#3-C(2)-H(2C) | 109.1 |
| C(2)-H(2C) | 0.99 | O(4)-Y(1)-O(5) | 71.88(10) | C(1)-C(2)-H(2D) | 109.1 |
| C(2)-H(2D) | 0.99 | O(4)#1-Y(1)-O(5) | 71.88(10) | C(1)#3-C(2)-H(2D) | 109.1 |
| O(4)-Y(1)-O(4)#1 | 115.57(19) | O(3)#1-Y(1)-O(5) | 80.04(11) | H(2C)-C(2)-H(2D) | 107.8 |
| O(4)-Y(1)-O(3)#1 | 142.45(11) | O(3)-Y(1)-O(5) | 80.04(11) |  |  |
| O(4)#1-Y(1)-O(3)#1 | 76.99(11) | O(2)-Y(1)-O(5) | 144.52(9) |  |  |
| Gd(C_3_H_2_O_4_)NO_3_·4H_2_O | | | | | |
| Gd(1)-O(4)#1 | 2.321(5) | O(4)-Gd(1)-O(3) | 76.06(16) | O(5)#2-Gd(1)-O(1) | 80.62(19) |
| Gd(1)-O(4) | 2.321(5) | O(4)#1-Gd(1)-O(3) | 76.06(15) | O(5)#3-Gd(1)-O(3) | 140.28(14) |
| Gd(1)-O(1) | 2.452(6) | O(4)#1-Gd(1)-O(2) | 141.51(19) | O(5)#2-Gd(1)-O(3) | 140.28(14) |
| Gd(1)-O(3) | 2.426(7) | O(4)#1-Gd(1)-O(2)#1 | 79.7(2) | O(5)#3-Gd(1)-O(2)#1 | 112.35(18) |
| Gd(1)-O(2)#1 | 2.424(5) | O(4)-Gd(1)-O(2) | 79.7(2) | O(5)#2-Gd(1)-O(2) | 112.35(18) |
| Gd(1)-O(2) | 2.424(5) | O(4)-Gd(1)-O(2)#1 | 141.51(19) | O(5)#3-Gd(1)-O(2) | 72.51(16) |
| Gd(1)-O(5)#2 | 2.381(5) | O(4)-Gd(1)-O(5)#2 | 142.93(19) | O(5)#2-Gd(1)-O(2)#1 | 72.51(16) |
| Gd(1)-O(5)#3 | 2.381(5) | O(4)#1-Gd(1)-O(5)#2 | 77.49(18) | O(5)#3-Gd(1)-O(5)#2 | 73.6(2) |
| O(4)-C(1) | 1.250(7) | O(4)-Gd(1)-O(5)#3 | 77.49(18) | C(1)-O(4)-Gd(1) | 143.0(4) |
| O(7)-N(1) | 1.273(9) | O(4)#1-Gd(1)-O(5)#3 | 142.93(19) | O(6)#1-N(1)-O(7) | 119.2(5) |
| O(6)-N(1) | 1.219(8) | O(3)-Gd(1)-O(1) | 117.8(2) | O(6)-N(1)-O(7) | 119.2(5) |
| C(2)-C(1) | 1.523(7) | O(2)#1-Gd(1)-O(1) | 144.53(14) | O(6)-N(1)-O(6)#1 | 121.5(10) |
| C(2)-C(1)#4 | 1.523(7) | O(2)-Gd(1)-O(1) | 144.53(14) | C(1)#4-C(2)-C(1) | 111.8(7) |
| C(1)-O(5) | 1.261(7) | O(2)#1-Gd(1)-O(3) | 74.01(17) | O(4)-C(1)-C(2) | 116.1(6) |
| O(4)#1-Gd(1)-O(4) | 115.6(3) | O(2)-Gd(1)-O(3) | 74.01(17) | O(4)-C(1)-O(5) | 124.7(6) |
| O(4)#1-Gd(1)-O(1) | 72.09(16) | O(2)-Gd(1)-O(2)#1 | 69.2(3) | O(5)-C(1)-C(2) | 119.3(6) |
| O(4)-Gd(1)-O(1) | 72.09(16) | O(5)#3-Gd(1)-O(1) | 80.62(19) | C(1)-O(5)-Gd(1)#5 | 132.3(4) |
| Lu(C_3_H_2_O_4_)NO_3_·4H_2_O | | | | | |
| Gd(1)-O(4)#1 | 2.321(5) | O(4)-Gd(1)-O(3) | 76.06(16) | O(5)#2-Gd(1)-O(1) | 80.62(19) |
| Gd(1)-O(4) | 2.321(5) | O(4)#1-Gd(1)-O(3) | 76.06(15) | O(5)#3-Gd(1)-O(3) | 140.28(14) |
| Gd(1)-O(1) | 2.452(6) | O(4)#1-Gd(1)-O(2) | 141.51(19) | O(5)#2-Gd(1)-O(3) | 140.28(14) |
| Gd(1)-O(3) | 2.426(7) | O(4)#1-Gd(1)-O(2)#1 | 79.7(2) | O(5)#3-Gd(1)-O(2)#1 | 112.35(18) |
| Gd(1)-O(2)#1 | 2.424(5) | O(4)-Gd(1)-O(2) | 79.7(2) | O(5)#2-Gd(1)-O(2) | 112.35(18) |
| Gd(1)-O(2) | 2.424(5) | O(4)-Gd(1)-O(2)#1 | 141.51(19) | O(5)#3-Gd(1)-O(2) | 72.51(16) |
| Gd(1)-O(5)#2 | 2.381(5) | O(4)-Gd(1)-O(5)#2 | 142.93(19) | O(5)#2-Gd(1)-O(2)#1 | 72.51(16) |
| Gd(1)-O(5)#3 | 2.381(5) | O(4)#1-Gd(1)-O(5)#2 | 77.49(18) | O(5)#3-Gd(1)-O(5)#2 | 73.6(2) |
| O(4)-C(1) | 1.250(7) | O(4)-Gd(1)-O(5)#3 | 77.49(18) | C(1)-O(4)-Gd(1) | 143.0(4) |
| O(7)-N(1) | 1.273(9) | O(4)#1-Gd(1)-O(5)#3 | 142.93(19) | O(6)#1-N(1)-O(7) | 119.2(5) |
| O(6)-N(1) | 1.219(8) | O(3)-Gd(1)-O(1) | 117.8(2) | O(6)-N(1)-O(7) | 119.2(5) |
| C(2)-C(1) | 1.523(7) | O(2)#1-Gd(1)-O(1) | 144.53(14) | O(6)-N(1)-O(6)#1 | 121.5(10) |
| C(2)-C(1)#4 | 1.523(7) | O(2)-Gd(1)-O(1) | 144.53(14) | C(1)#4-C(2)-C(1) | 111.8(7) |
| C(1)-O(5) | 1.261(7) | O(2)#1-Gd(1)-O(3) | 74.01(17) | O(4)-C(1)-C(2) | 116.1(6) |
| O(4)#1-Gd(1)-O(4) | 115.6(3) | O(2)-Gd(1)-O(3) | 74.01(17) | O(4)-C(1)-O(5) | 124.7(6) |
| O(4)#1-Gd(1)-O(1) | 72.09(16) | O(2)-Gd(1)-O(2)#1 | 69.2(3) | O(5)-C(1)-C(2) | 119.3(6) |
| O(4)-Gd(1)-O(1) | 72.09(16) | O(5)#3-Gd(1)-O(1) | 80.62(19) | C(1)-O(5)-Gd(1)#5 | 132.3(4) |

#1-x-1, y, z #2-x-1/2, -y, z-1/2 #3-x, y, z #4-x-1/2, -y, z+1/2

**Table S6.** Interaction energies between NO_3_^-^ and H_2_O fragments in RE(C_3_H_2_O_4_)NO_3_·4H_2_O (RE=Y, Gd, Lu) calculated by sobEDAw.

| **Energy**  **（kcal/mol）** | **YCN** | | **GCN** | | **LCN** | |
| --- | --- | --- | --- | --- | --- | --- |
|  | **N-O_1_···H** | **N-O_2_···H** | **N-O_1_···H** | **N-O_2_···H** | **N-O_1_···H** | **N-O_2_···H** |
|  | **1.825 Å** | **2.159 Å** | **1.825 Å** | **2.030 Å** | **1.929 Å** | **2.227 Å** |
| **Electrostatic** | -16.64 | -7.27 | -15.61 | -8.60 | -12.79 | -11.31 |
| **Exchange** | 14.98 | 8.03 | 15.26 | 11.58 | 11.10 | 10.50 |
| **Induction** | -6.11 | -2.12 | -6.04 | -4.06 | -3.98 | -3.02 |
| **Dispersion** | -4.02 | -3.43 | -4.08 | -3.96 | -3.58 | -4.04 |
| **Total** | -11.79 | -4.79 | -10.46 | -5.04 | -9.25 | -7.88 |
| **Strength Level** | Ⅲ | Ⅱ | Ⅱ | Ⅱ | Ⅱ | Ⅱ |

# Strength level classification: Ⅰ (Very week)— > -2.5 kcal/mol; Ⅱ (Weak to medium)— -2.5 to -14.0 kcal/mol; Ⅲ (Medium)— -11.0 to -15.0 kcal/mol; Ⅳ (Strong)— < -15.0 kcal/mol.

**Table S7.** Atomic or functional groups contributions to the SHG coefficients based on the Bader charge analysis.

| **_Contribution_ ^Compound^** | **YCN** | **GCN** | **LCN** |
| --- | --- | --- | --- |
| **C_3_H_2_O_4_^2-^ (%)** | 42.25 | 41.93 | 42.83 |
| **NO_3_^-^ (%)** | 42.86 | 40.75 | 44.58 |
| **RE^3+^ (%)** | 4.22 | 9.01 | 4.09 |
| **H_2_O (%)** | 10.66 | 8.31 | 8.50 |

**Table S8.** Linear and NLO properties of Nitrate Crystals.

| No. | Formula | Space group | Birefringence | *d*_ij_ (pm/V) (1064 nm) | PSHG (🞩KDP) | Ref. |
| --- | --- | --- | --- | --- | --- | --- |
| 1 | NH[C(NH_2_)_2_]_2_(NO_3_)_2_ | *Pca*2_1_ | 0.122 at 550 nm | *d*_32_= 0.87 | 3.5 | ^[15]^ |
| 2 | [C(NH_2_)_2_NHNO_2_][C(NH_2_)_3_](NO_3_)_2_ | *Cc* | 0.071 at 550 nm | *d*_33_= -0.84 | 1.5 | ^[16]^ |
| 3 | *α*-(C_2_H_5_N_4_)(NO_3_) | *P*1 | 0.069 at 546 nm | *d*_eff_= 0.91 | 3.5 | ^[17]^ |
| 4 | *β*-(C_2_H_5_N_4_)(NO_3_) | *P*2_1_ | 0.046 at 546 nm | *d*_eff_= 0.03 | 1.0 |  |
| 5 | KNO_3_SO_3_NH_3_ | *P*2_1_ | 0.096 at 546 nm | *d*_21_= 4.38 | 10.0 | ^[18]^ |
| 6 | RbNO_3_SO_3_NH_3_ | *Pmc*2_1_ | 0.070 at 546 nm | *d*_31_= -3.27 | 7.0 | ^[19]^ |
| 7 | Rb_2_Na(NO_3_)_3_ | *Pmc*2_1_ | / | *d*_15_= 3.48 | 5.0 | ^[20]^ |
| 8 | Sr_2_(OH)_3_NO_3_ | *P*$\bar{6}$2*m* | / | / | 3.6 | ^[21]^ |
| 9 | Sr(NH_2_SO_3_)(NO_3_)·H_2_O | *Pca*2_1_ | 0.077 at 546.1 nm | *d*_33_= 4.28 | 5.2 | ^[22]^ |
| 10 | Sr(NO_3_)(NH_2_SO_3_)·H_2_O | *Pca*2_1_ | 0.067 at 532 nm | */* | 5.1 | ^[23]^ |
| 11 | Ba_2_NO_3_(OH)_3_ | *P*$\bar{6}$2*m* | 0.080 at 532 nm | *d*_22_= 2.41 | 4.0 | ^[24]^ |
| 12 | Y(OH)_2_NO_3_ | *P*2_1_ | 0.133 at 589.6 nm | *d*_23_= 1.02 | 5.6 | ^[25]^ |
| 13 | La(OH)_2_NO_3_ | *P*2_1_ | 0.146 at 589.6 nm | *d*_23_= 0.95 | 5.0 |  |
| 14 | Gd(OH)_2_NO_3_ | *P*2_1_ | 0.112 at 589.6 nm | *d*_23_= 1.40 | 5.5 |  |
| 15 | K_2_La(NO_3_)_5_·2H_2_O | *Fdd*2 | 0.067 at 546.1 nm | */* | 3.0 | ^[26]^ |
| 16 | K_2_Ce(NO_3_)_5_·2H_2_O | *Fdd*2 | 0.069 at 546.1 nm | */* | 3.0 |  |
| 17 | Na_10_Zn(NO_3_)_4_(SO_3_S)_4_ | *P*$\bar{4}$ | 0.013 at 550 nm | / | 1.2 | ^[27]^ |
| 18 | K_2_Hg(NO_3_)_4_ | $I\bar{4}$2*m* | 0.107 at 546 nm | *d*_36_= 3.31 | 9.2 | ^[28]^ |
| 19 | Rb_2_Hg(NO_3_)_4_ | $I\bar{4}$2*m* | 0.092 at 546 nm | *d*_36_= 3.08 | 8.8 |  |
| 20 | [Pb_4_(OH)_4_](NO_3_)_4_ | *Cc* | / | / | 0.7 | ^[29]^ |
| 21 | Pb_2_BO_3_NO_3_ | *P*6_3_*mc* | 0.174 at 1064 nm | *d*_32_= 4.43 | 9.0 | ^[30]^ |
| 22 | Pb_2_(NO_3_)_2_(H_2_O)F_2_ | *Amm*2 | 0.23 at 1064 nm | *d*_31_= 4.55 | 12.0 | ^[31]^ |
| 23 | Pb_2_(SeO_3_)(NO_3_)_2_ | *Pmn*2_1_ | / | / | 2.0 | ^[32]^ |
| 24 | [LaPb_8_O(OH)_10_(H_2_O)](NO_3_)_7_ | *Cc* | / | */* | 1.3 | ^[33]^ |
| 25 | [LaPb_8_O(OH)_10_(H_2_O)](NO_3_)_7_·2H_2_O | *P*2_1_2_1_2_1_ | / | */* | 1.1 |  |
| 26 | Pb_16_(OH)_16_(NO_3_)_16_ | *Cc* | / | / | 3.5 | ^[34]^ |
| 27 | Bi_6_O_6_F_5_(NO_3_) | *R*3 | / | / | 3.0 | ^[35]^ |
| 28 | Bi_2_O_2_[NO_2_(OH)] | *Cmc*2_1_ | 0.045 at 1064 nm | *d*_33_= 3.24 | 6.0 | ^[36]^ |
| 29 | Bi_3_TeO_6_OH(NO_3_)_2_ | *P*2_1_ | 0.115 at 1064 nm | *d*_16_ = 1.31 | 3.0 | ^[37]^ |
| 30 | Y(C_3_H_2_O_4_)NO_3_·4H_2_O | *Pmn*2_1_ | 0.135 at 589.3 nm | *d*_15_= 4.48 | 9.5 | This work |
| 31 | Gd(C_3_H_2_O_4_)NO_3_·4H_2_O | *Pmn*2_1_ | 0.134 at 589.3 nm | *d*_15_= 4.05 | 8.8 |  |
| 32 | Lu(C_3_H_2_O_4_)NO_3_·4H_2_O | *Pmn*2_1_ | 0.135 at 589.3 nm | *d*_15_= 3.77 | 8.5 |  |

**Table S9.** Linear and NLO properties of Malonate Crystals and their derivatives.

| No. | Formula | Space group | Birefringence | *d*_ij_ (pm/V) (1064 nm) | PSHG (🞩KDP) | Ref. |
| --- | --- | --- | --- | --- | --- | --- |
| 1 | KLi(C_3_H_2_O_4_)·H_2_O | *Pna*2_1_ | 0.103 at 1064 nm | *d*_33_= 1.91 | 3.0 | ^[38]^ |
| 2 | NH_4_[LiC_3_H(CH_3_)O_4_] | *P*2_1_ | 0.060 at 546 nm | *d*_21_= -2.01 | 4.0 | ^[39]^ |
| 3 | K_2_(C_3_F_2_O_4_) | *Cmc*2_1_ | 0.168 at 546 nm | *d*_31_= 1.06 | 3.6 | ^[40]^ |
| 4 | Rb_2_(C_3_F_2_O_4_)·2H_2_O | *Fdd*2 | 0.098 at 546 nm | *d*_31_= -2.50 | 5.5 |  |
| 5 | Cs_2_(C_3_F_2_O_4_)·2H_2_O | *Fdd*2 | 0.073 at 546 nm | *d*_31_= -2.45 | 5.7 |  |
| 6 | (NH_4_)_2_(C_3_H(OH)O_4_) | *Pna*2_1_ | 0.119 at 546 nm | *d*_33_= 1.93 | 5.3 |  |
| 7 | Li_2_(C_3_HFO_4_)·H_2_O | *Pna*2_1_ | 0.074 at 546 nm | *d*_33_= 2.25 | 2.3 |  |
| 8 | [Li_2_C_3_(CH_3_)_2_O_4_]_2_·3H_2_O | *Fdd*2 | 0.071 at 514 nm | *d*_32_= 2.87 | 6.5 |  |
| 9 | Mg(C_3_O_4_H_2_)(H_2_O)_2_ | *Pca*2_1_ | 0.164 at 546.1 nm | *d*_31_= 0.88 | 3.0 | ^[41]^ |
| 10 | Li_3_[Be_3_(OH)_3_(C_3_H_2_O_4_)_3_]·7H_2_O | *R*3*c* | 0.067 at 589.3 nm | *d*_33_= 1.42 | 4.0 | ^[42]^ |
| 11 | C(NH_2_)_3_C_3_H_3_O_4_ | *Ccc*2 | 0.122 at 546.1 nm | *d*_33_= 1.24 | 2.0 | ^[43]^ |
| 12 | C(NH_2_)_3_C_4_H_5_O_4_ | *Ccc*2 | 0.159 at 546.1 nm | *d*_33_= 0.52 | 1.0 |  |
| 13 | Rb_2_[(C_3_H_2_O_4_)(H_3_BO_3_)] | *P*2_1_2_1_2_1_ | 0.053 at 546.1 nm | *d*_14_= -0.45 | 1.1 | ^[44]^ |
| 14 | Cs_2_[(C_3_H_2_O_4_)(H_3_BO_3_)]⋅0.26H_2_O | *P*2_1_2_1_2_1_ | 0.055 at 546.1 nm | *d*_14_= -0.19 | 1.2 |  |
| 15 | K_1_.1Rb_0_.9[(C_3_H_2_O_4_)(H_3_BO_3_)] | *P*2_1_2_1_2_1_ | 0.046 at 546.1 nm | *d*_14_= -0.25 | 0.5 |  |
| 16 | K_1_.1Cs_0_.9[(C_3_H_2_O_4_)(H_3_BO_3_)] | *P*2_1_2_1_2_1_ | 0.053 at 546.1 nm | *d*_14_= 0.39 | 0.6 |  |
| 17 | RbLi(C_3_H_2_O_4_)·H_2_O | *Pna*2_1_ | 0.163 at 546 nm | */* | 3.0 | ^[45]^ |
| 18 | Rb_2_Li(C_3_H_3_O_4_)_3_·H_2_O | *P*2_1_ | 0.043 at 546 nm | */* | 1.5 |  |
| 19 | Y(C_3_H_2_O_4_)NO_3_·4H_2_O | *Pmn*2_1_ | 0.135 at 589.3 nm | *d*_15_= 4.48 | 9.5 | This work |
| 20 | Gd(C_3_H_2_O_4_)NO_3_·4H_2_O | *Pmn*2_1_ | 0.134 at 589.3 nm | *d*_15_= 4.05 | 8.8 |  |
| 21 | Lu(C_3_H_2_O_4_)NO_3_·4H_2_O | *Pmn*2_1_ | 0.135 at 589.3 nm | *d*_15_= 3.77 | 8.5 |  |

**
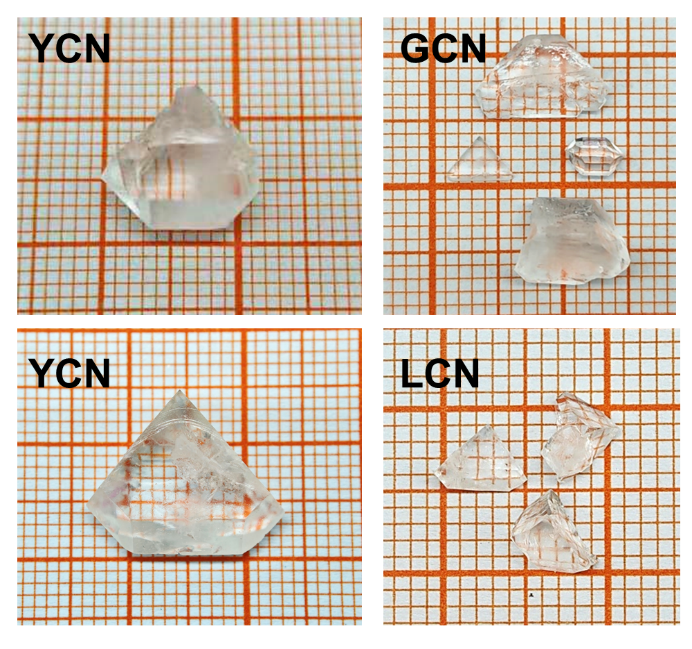
**

**Figure S1**. Singel crystal photos of YCN, GCN, and LCN.


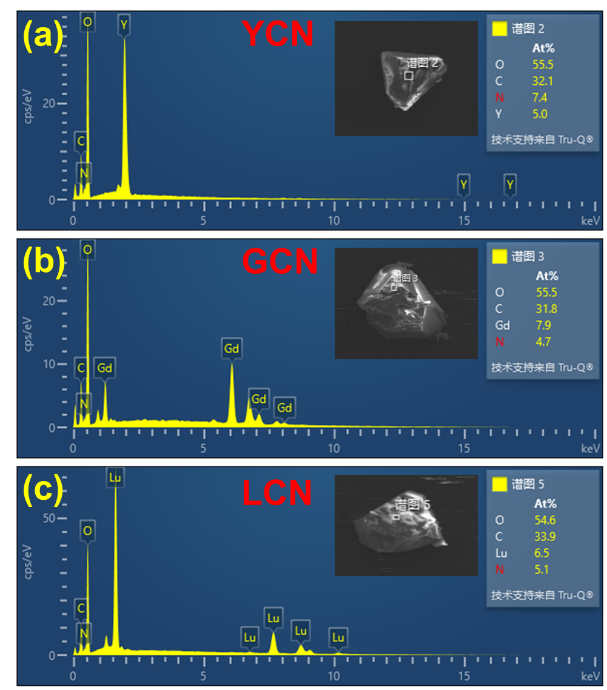


**Figure S2**. Energy dispersive X-ray spectroscopies of YCN, GCN, and LCN.


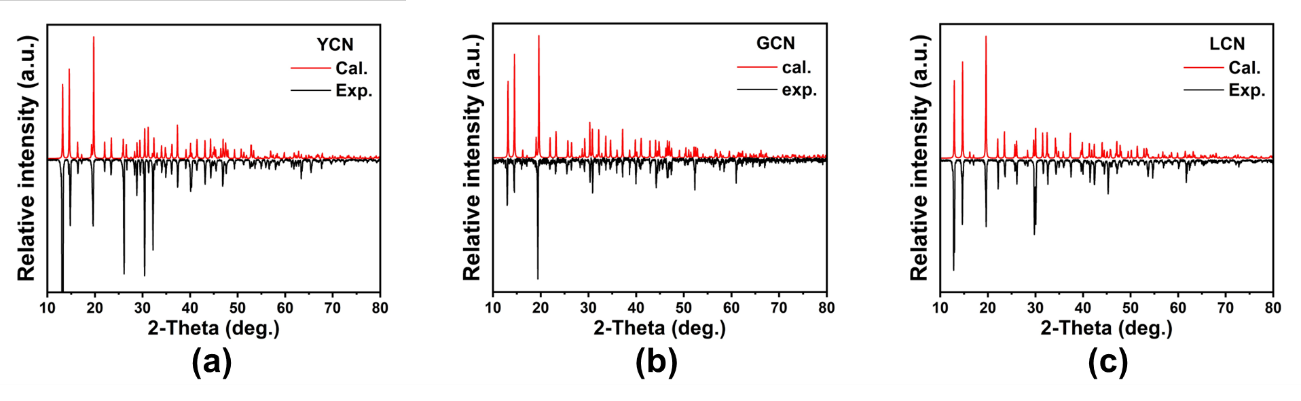


**Figure S3**. Powder Xray diffraction (XRD) patterns of YCN, GCN, and LCN.


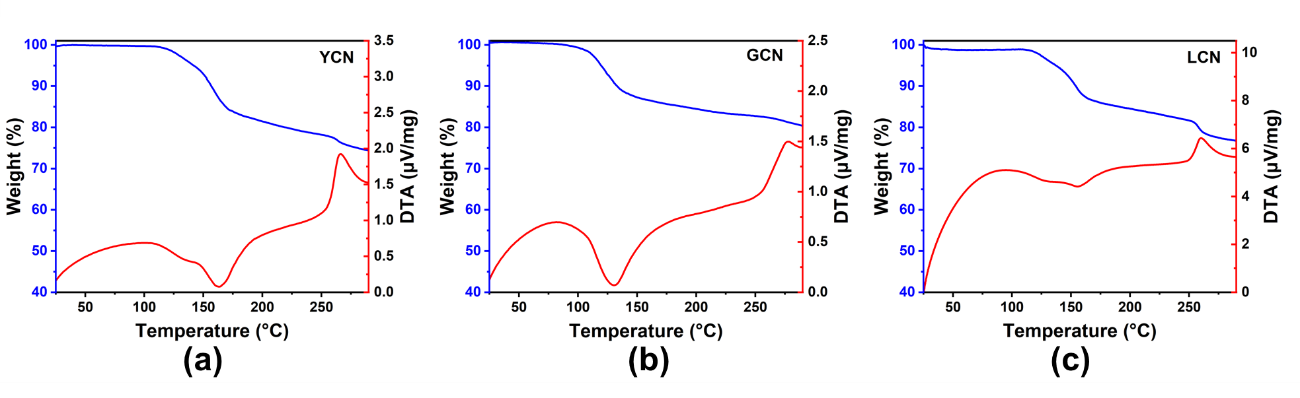


**Figure S4**. TG-DTA curves of YCN, GCN, and LCN.


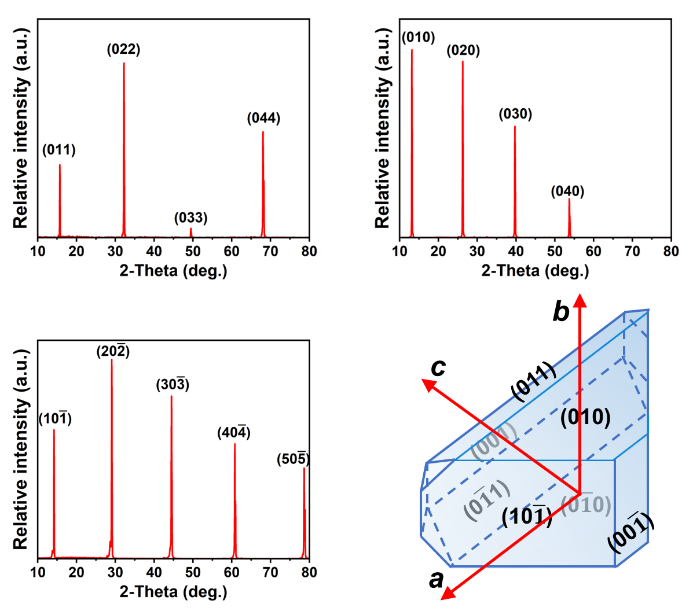


**Figure S5**. XRD patterns of the natural growth facets of YCN and its simulation diagram.


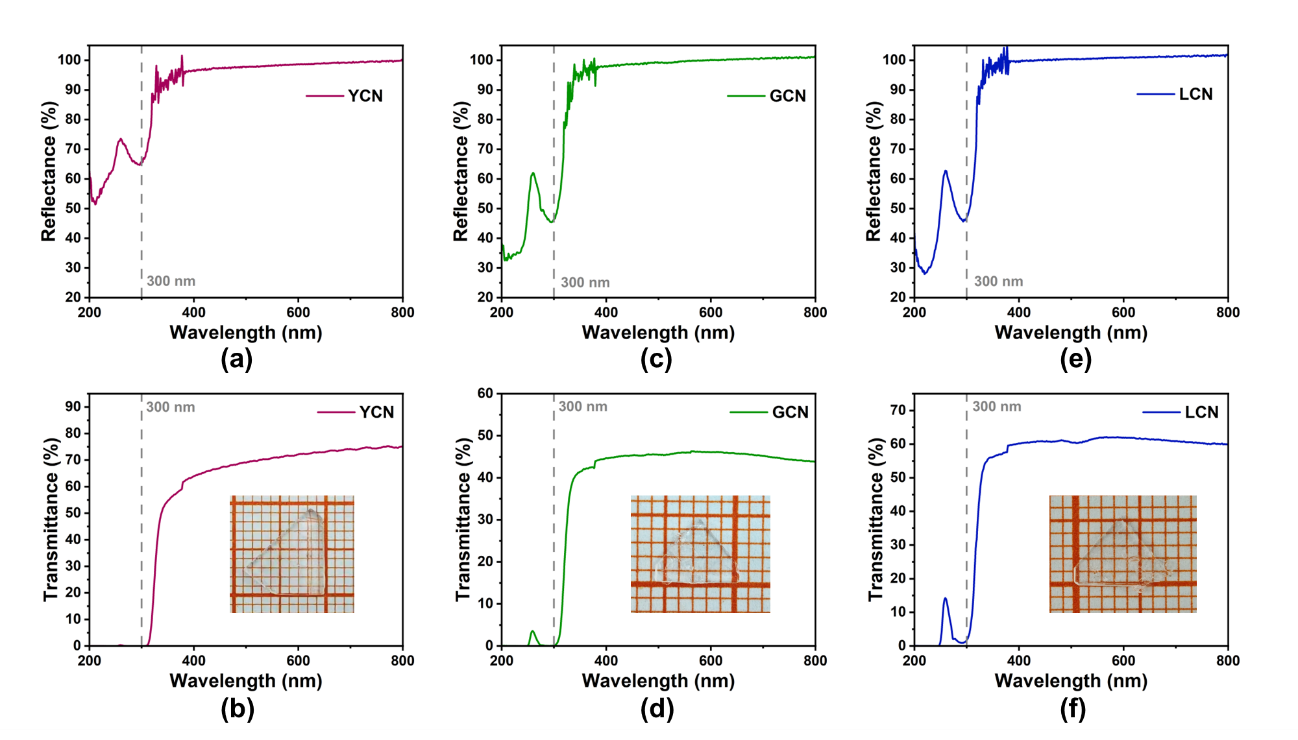


**Figure S6**. (a, c, e) The UV/Vis diffuse-reflectance spectroscopies of YCN, GCN, and LCN; (b, d, f) UV–Vis–NIR transmission spectrums of YCN, GCN, and LCN.


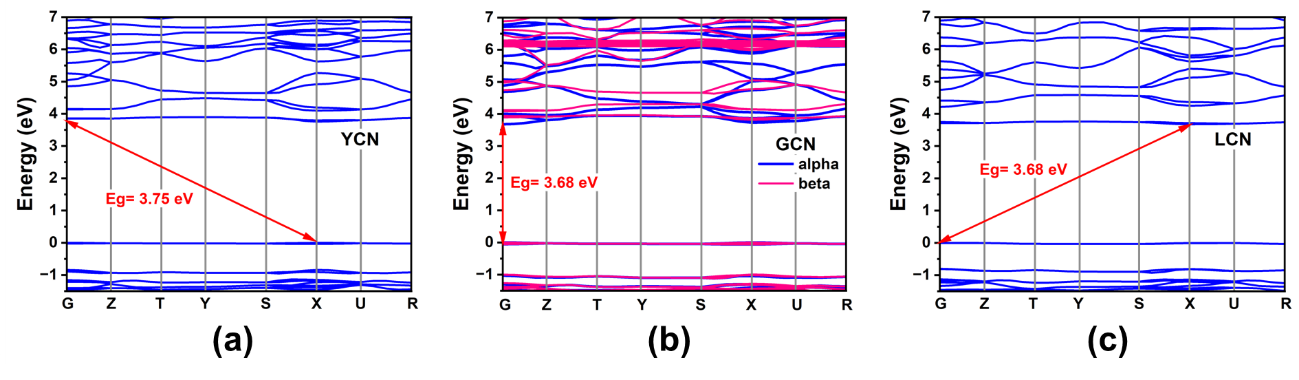


**Figure S7**. Calculated electronic band structure for YCN, GCN, and LCN.


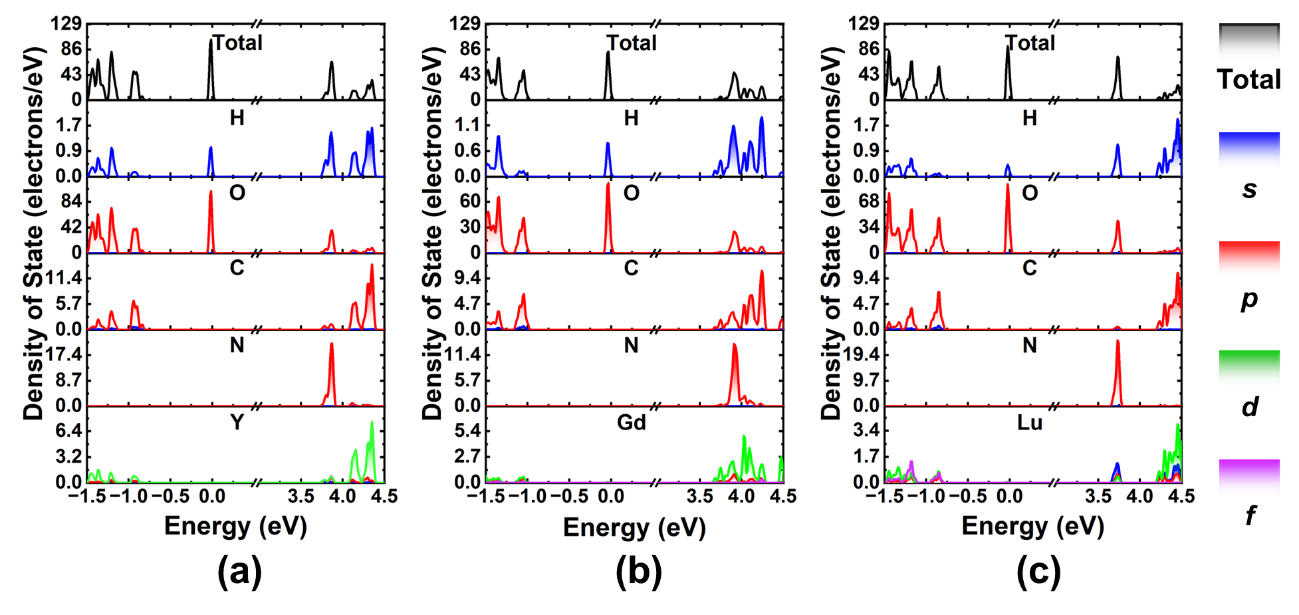


**Figure S8**. Partial density of states (PDOS) curves for YCN, GCN, and LCN.


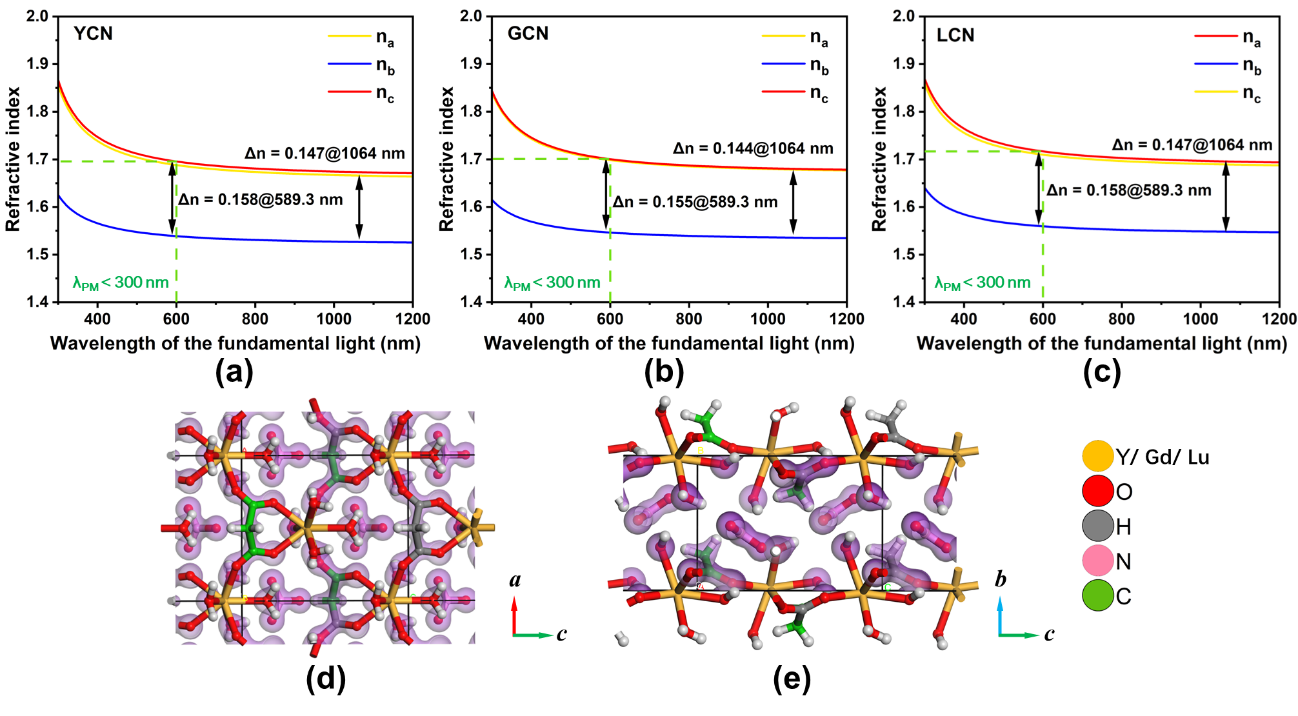


**Figure S9**. (a-c) Calculated refractive indices for YCN, GCN, and LCN; d-e) the electron density maps for YCN, GCN, and LCN.


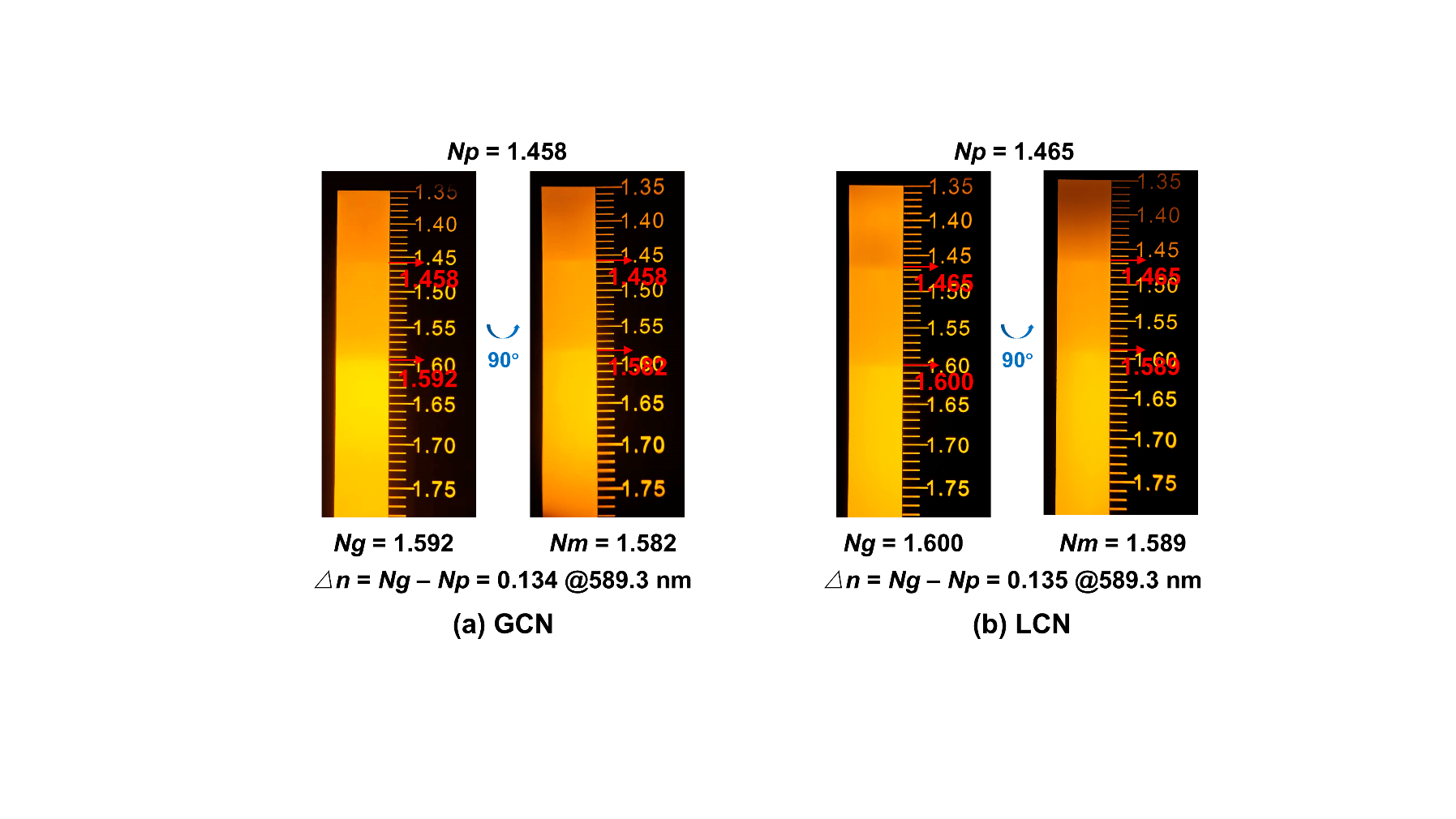


**Figure S10**. The corresponding experimental refractive indices for GCN and LCN through their (010) facets.


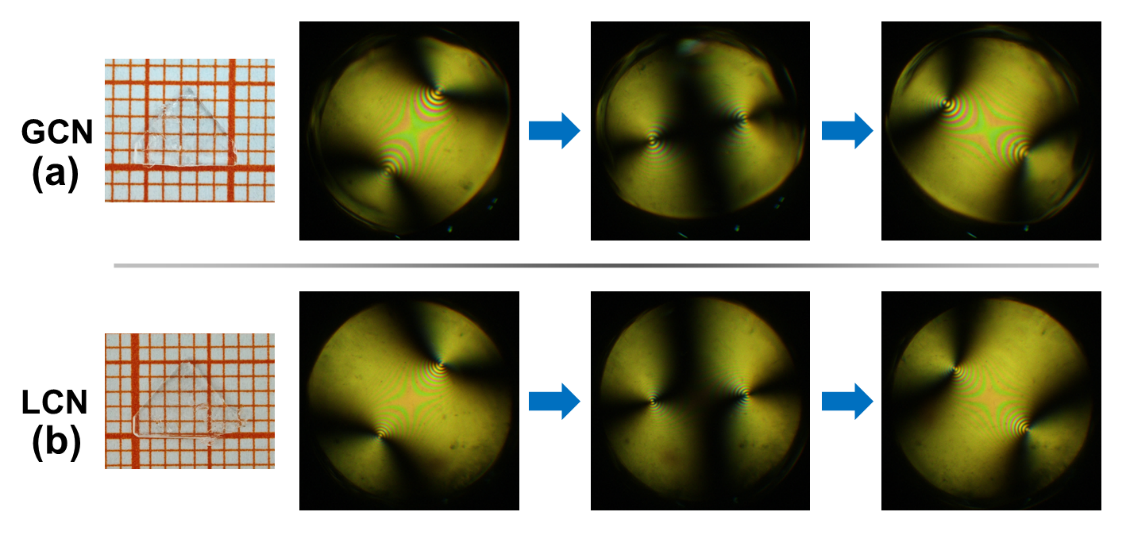


**Figure S11**. The conoscopic interference patterns of (010) facets for GCN, and LCN.


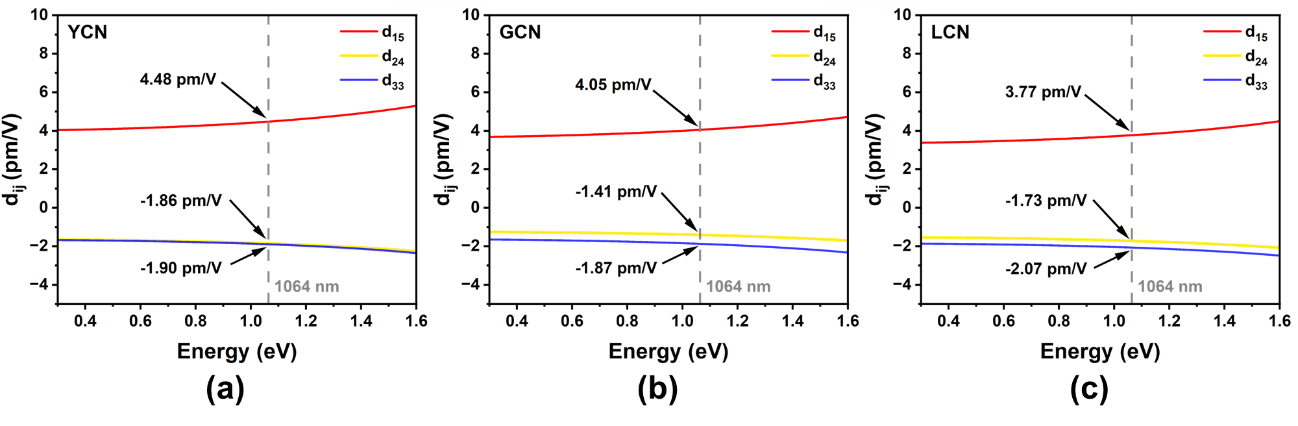


**Figure S12**. The calculated SHG coefficients for YCN, GCN, and LCN.


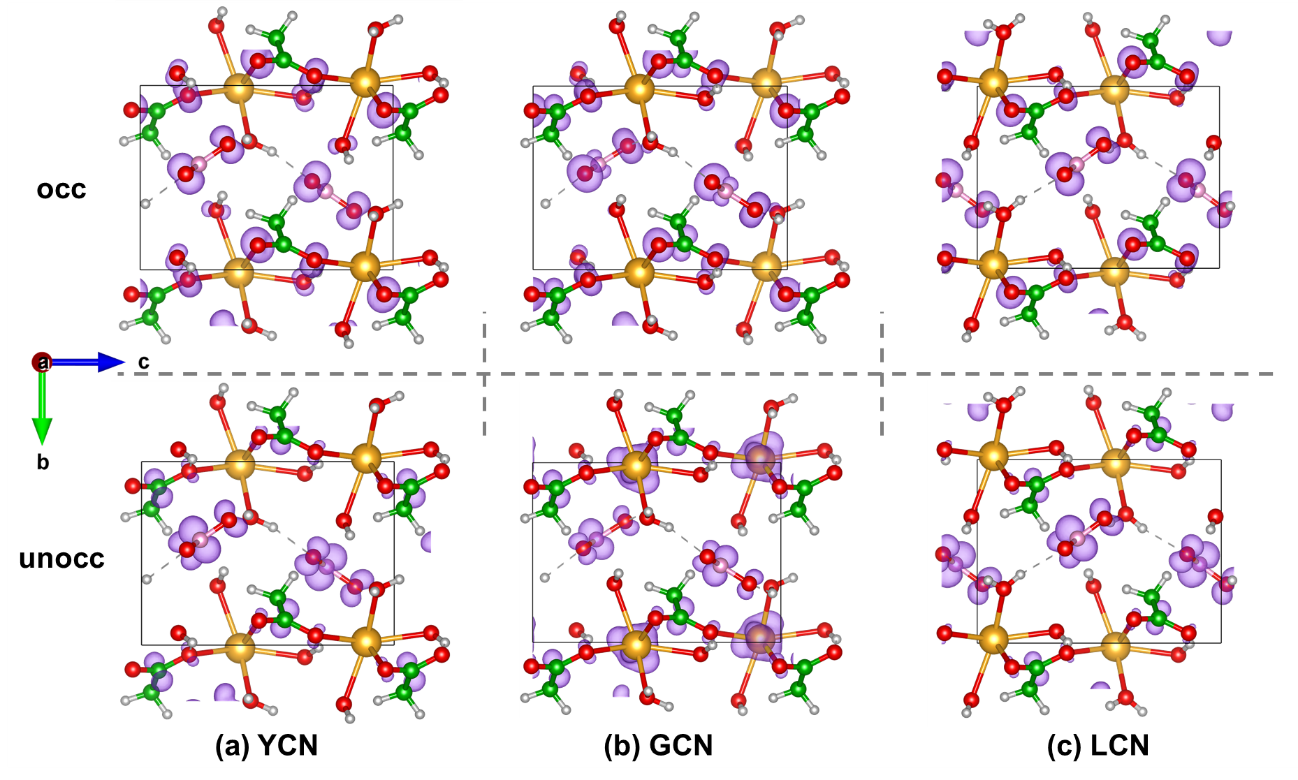


**Figure S13**. Calculated SHG density maps of *d*_31_ in occupied and unoccupied states for YCN, GCN, and LCN.

**References**

[1] G. M. Sheldrick, “*SHELXT* – integrated space-group and crystal-structure determination” *Acta Crystallogr., Sect. A: Found. Adv.* **71**, (2015): 3–8.

[2] S. K. Kurtz, T. T. Perry, “A powder technique for the evaluation of nonlinear optical materials” *J. Appl. Phys.* **39**, (1968): 3798–3813.

[3] S. J. Cyvin, J. E. Rauch, J. C. Decius, “Theory of hyper-raman effects (nonlinear inelastic light scattering): Selection rules and depolarization ratios for the second-order polarizability” *J. Chem. Phys.* **43**, (1965): 4083–4095.

[4] A. M. Rappe, K. M. Rabe, E. Kaxiras, J. D. Joannopoulos, “Optimized pseudopotentials” *Phys. Rev. B* **41**, (1990): 1227–1230.

[5] J. Behler, K. Reuter, M. Scheffler, “Behler, reuter, and scheffler reply:” *Phys. Rev. Lett.* **96**, (2006): 79802.

[6] C. S. Wang, B. M. Klein, “First-principles electronic structure of si, ge, GaP, GaAs, ZnS, and ZnSe. I. Self-consistent energy bands, charge densities, and effective masses” *Phys. Rev. B* **24**, (1981): 3393–3416.

[7] R. W. Godby, M. Schlüter, L. J. Sham, “Self-energy operators and exchange-correlation potentials in semiconductors” *Phys. Rev. B* **37**, (1988): 10159–10175.

[8] D. J. Moss, E. Ghahramani, J. E. Sipe, H. M. Van Driel, “Band-structure calculation of dispersion and anisotropy in χ → ( 3 ) for third-harmonic generation in si, ge, and GaAs” *Phys. Rev. B* **41**, (1990): 1542–1560.

[9] W.-D. Cheng, C.-S. Lin, H. Zhang, G.-L. Chai, “Theoretical evaluation on terahertz source generators from ternary metal chalcogenides of PbM_6_Te_10_ (M = Ga, In)” *J. Phys. Chem. C* **122**, (2018): 4557–4564.

[10] M.-H. Lee, C.-H. Yang, J.-H. Jan, “Band-resolved analysis of nonlinear optical properties of crystalline and molecular materials” *Phys. Rev. B* **70**, (2004): 235110.

[11] S. Emamian, T. Lu, H. Kruse, H. Emamian, “Exploring Nature and Predicting Strength of Hydrogen Bonds: A Correlation Analysis Between Atoms‐in‐Molecules Descriptors, Binding Energies, and Energy Components of Symmetry‐Adapted Perturbation Theory” *J. Comput. Chem.* **40**, (2019): 2868–2881.

[12] Z. Y. Liu, T. Lu, Q. X. Chen, “Intermolecular interaction characteristics of the all-carboatomic ring, cyclo[18]carbon: Focusing on molecular adsorption and stacking” *Carbon* **171**, (2021): 514–523.

[13] T. Lu, F. W. Chen, “Multiwfn: A multifunctional wavefunction analyzer” *J. Comput. Chem.* **33**, (2012): 580–592.

[14] T. Lu, “A comprehensive electron wavefunction analysis toolbox for chemists, multiwfn” *J. Chem. Phys.* **161**, (2024): 82503.

[15] Y. Q. Zhang, Q. R. Ding, Y. Q. Li, X. Y. Song, W. Q. Huang, Y. Zhou, Q. P. Shao, Z. Y. Bai, S. G. Zhao, J. H. Luo, “Exploiting rigid inorganic π-conjugated units and deprotonatable organic π-conjugated dimers for enhanced linear and nonlinear optical properties” *Laser Photonics Rev.* **19**, (2025): 2500368.

[16] D. Yan, M.-M. Ren, Q. Liu, F.-F. Mao, Y. Ma, R.-L. Tang, H. B. Huang, B. B. Zhang, X.-D. Zhang, S.-F. Li, “[C(NH_2_)_2_NHNO_2_ ][C(NH_2_)_3_](NO_3_)_2_ : A mixed organic cationic hybrid nitrate with an unprecedented nonlinear-optical-active unit” *Inorg. Chem.* **62**, (2023): 4757–4761.

[17] Q. Xia, X. X. Jiang, C. B. Jiang, H. J. Zhang, Y. L. Hu, L. Qi, C. Wu, G. F. Wei, Z. S. Lin, Z. P. Huang, M. G. Humphrey, C. Zhang, “pH‐dependent switching between nonlinear‐optical‐active nitrate‐based supramolecular polymorphs” *Angew. Chem. Int. Ed.* **64**, (2025): e202503136.

[18] Haotian. Tian, C. S. Lin, X. Zhao, S. H. Fang, H. Li, C. Wang, N. Ye, M. Luo, “Design of a new ultraviolet nonlinear optical material KNO_3_SO_3_NH_3_ exhibiting an unexpected strong second harmonic generation response” *Mater. Today Phys.* **28**, (2022): 100849.

[19] Y. X. Song, C. S. Lin, X. Zhao, T. Yan, N. Ye, H. T. Tian, M. Luo, “Synergistic combination of different types of functional motif in Rb(NO_3_)(SO_3_NH_3_) for realizing excellent ultraviolet optical nonlinearity” *Inorg. Chem. Front.* **11**, (2024): 4329–4335.

[20] G. H. Zou, C. S. Lin, H. Kim, H. Jo, K. Ok, “Rb_2_Na(NO_3_)_3_: A congruently melting UV-NLO crystal with a very strong second-harmonic generation response” *Crystals* **6**, (2016): 42.

[21] L. Huang, G. H. Zou, H. Q. Cai, S. C. Wang, C. S. Lin, N. Ye, “Sr_2_(OH)_3_NO_3_: The first nitrate as a deep UV nonlinear optical material with large SHG responses” *J. Mater. Chem. C* **3**, (2015): 5268–5274.

[22] X. Hao, C. S. Lin, M. Luo, N. Ye, “Sr(NH_2_SO_3_)(NO_3_)·H_2_O: An ultraviolet nonlinear optical material exhibiting strong second-harmonic generation response and sufficient birefringence” *Inorg. Chem.* **62**, (2023): 18020–18024.

[23] X. F. Wang, Y. Li, Z. L. Chen, J. Lee, F. F. Zhang, K. R. Poeppelmeier, S. L. Pan, K. M. Ok, “Sr(NO_3_)(NH_2_SO_3_)·H_2_O: first nitrate sulfamate revealing remarkable second harmonic generation and optimized birefringence” *Small Struct.* **4**, (2023): 2300274.

[24] X. H. Dong, L. Huang, Q. Y. Liu, H. M. Zeng, Z. E. Lin, D. G. Xu, G. H. Zou, “Perfect balance harmony in Ba_2_NO_3_(OH)_3_: A beryllium-free nitrate as a UV nonlinear optical material” *Chem. Commun.* **54**, (2018): 5792–5795.

[25] Y. X. Song, M. Luo, C. S. Lin, N. Ye, “Structural modulation of nitrate group with cations to affect SHG responses in RE(OH)_2_NO_3_ (RE = La, Y, and Gd): New polar materials with large NLO effect after adjusting pH values of reaction systems” *Chem. Mater.* **29**, (2017): 896–903.

[26] C. A. Ebbers, L. D. DeLoach, M. Webb, D. Eimerl, S. P. Velsko, D. A. Keszler, “Nonlinear optical properties of K_2_La(NO_3_)_5_·2H_2_O and K_2_Ce(NO_3_)_5_·2H_2_O” *IEEE J. Quantum Electron.* **29**, (1993): 497–507.

[27] Z. H. Yu, Q. R. Ding, Y. H. Jiang, W. Q. Huang, C. S. Yang, S. G. Zhao, J. H. Luo, “Na_10_Zn(NO_3_)_4_(SO_3_S)_4_ : A nonlinear optical crystal combining inorganic π-conjugated and non-π-conjugated heteroanion groups” *Inorg. Chem. Front.* **11**, (2024): 107–113.

[28] L. Qi, X. X. Jiang, K. N. Duanmu, C. Wu, Z. S. Lin, Z. P. Huang, M. G. Humphrey, C. Zhang, “Quadruple-bidentate nitrate-ligated A_2_Hg(NO_3_)_4_ (A=K, Rb): Strong second-harmonic generation and sufficient birefringence” *Angew. Chem. Int. Ed.* **62**, (2023): e202309365.

[29] G. X. Wang, M. Luo, N. Ye, C. S. Lin, W. D. Cheng, “Series of lead oxide hydroxide nitrates obtained by adjusting the pH values of the reaction systems” *Inorg. Chem.* **53**, (2014): 5222–5228.

[30] J.-L. Song, C.-L. Hu, X. Xu, F. Kong, J.-G. Mao, “A facile synthetic route to a new SHG material with two types of parallel π-conjugated planar triangular units” *Angew. Chem.* **127**, (2015): 3750–3753.

[31] G. Peng, Y. Yang, Y.-H. Tang, M. Luo, T. Yan, Y. Q. Zhou, C. S. Lin, Z. S. Lin, N. Ye, “Collaborative enhancement from Pb^2+^ and F^−^ in Pb_2_(NO_3_)_2_(H_2_O)F_2_ generates the largest second harmonic generation effect among nitrates” *Chem. Commun.* **53**, (2017): 9398–9401.

[32] C.-Y. Meng, L. Geng, W.-T. Chen, M.-F. Wei, K. Dai, H.-Y. Lu, W.-D. Cheng, “Syntheses, structures, and characterizations of a new second-order nonlinear optical material: Pb_2_(SeO_3_)(NO_3_)_2_” *J. Alloys Compd.* **640**, (2015): 39–44.

[33] G. X. Wang, M. Luo, C. S. Lin, N. Ye, Y. Q. Zhou, W. D. Cheng, “Lanthanum lead oxide hydroxide nitrates with a nonlinear optical effect” *Inorg. Chem.* **53**, (2014): 12584–12589.

[34] L. X. Chang, L. Wang, X. Su, S. L. Pan, R. S. L. T. Hailili, H. W. Yu, Z. H. Yang, “A nitrate nonlinear optical crystal Pb_16_(OH)_16_(NO_3_)_16_ with a large second-harmonic generation response” *Inorg. Chem.* **53**, (2014): 3320–3325.

[35] E. J. Cho, S.-J. Oh, H. Jo, J. S. Lee, T.-S. You, K. M. Ok, “Layered bismuth oxyfluoride nitrates revealing large second-harmonic generation and photocatalytic properties” *Inorg. Chem.* **58**, (2019): 2183–2190.

[36] R. H. Cong, T. Yang, F. H. Liao, Y. X. Wang, Z. S. Lin, J. H. Lin, “Experimental and theoretical studies of second harmonic generation for Bi_2_O_2_[NO_3_(OH)]” *Mater. Res. Bull.* **47**, (2012): 2573–2578.

[37] S. G. Zhao, Y. Yang, Y. G. Shen, B. Q. Zhao, L. N. Li, C. M. Ji, Z. Y. Wu, D. Q. Yuan, Z. S. Lin, M. C. Hong, J. H. Luo, “Cooperation of three chromophores generates the water-resistant nitrate nonlinear optical material Bi_3_TeO_6_OH(NO_3_)_2_” *Angew. Chem.* **129**, (2017): 555–559.

[38] L. L. Cao, H. T. Tian, D. H. Lin, C. S. Lin, F. Xu, Y. L. Han, T. Yan, J. D. Chen, B. X. Li, N. Ye, M. Luo, “A flexible functional module to regulate ultraviolet optical nonlinearity for achieving a balance between a second-harmonic generation response and birefringence” *Chem. Sci.* **13**, (2022): 6990–6997.

[39] H. T. Tian, C. S. Lin, Y. Q. Zhou, X. Zhao, H. X. Fan, T. Yan, N. Ye, M. Luo, “Design of the ionic organic nonlinear optical material NH_4_[LiC_3_H(CH_3_)O_4_] with ultrawide band gap and moderate birefringence” *Angew. Chem. Int. Ed.* **62**, (2023): e202304858.

[40] H. T. Tian, C. S. Lin, B. X. Li, X. Zhao, T. Yan, N. Ye, M. Luo, “Designing strong polarity and high configurational entropy flexible units toward excellent ultraviolet nonlinear optical materials” *Adv. Funct. Mater.* **34**, (2024): 2402295.

[41] L. L. Wu, C. S. Lin, H. T. Tian, Y. Q. Zhou, H. X. Fan, S. D. Yang, N. Ye, M. Luo, “Mg(C_3_O_4_H_2_)(H_2_O)_2_ : A new ultraviolet nonlinear optical material derived from KBe_2_BO_3_F_2_ with high performance and excellent water-resistance” *Angew. Chem. Int. Ed.* **63**, (2024): e202315647.

[42] L. L. Wu, C. S. Lin, H. T. Tian, T. Yan, B.-X. Li, H. X. Fan, Y. J. Fan, S. D. Yang, M. Luo, “Engineering an excellent *β* -BaB_2_O_4_ -inspired UV nonlinear optical material through secondary building unit substitution” *Angew Chem Int Ed* **64**, (2025): e202500877.

[43] L. L. Wu, C. S. Lin, H. X. Fan, S. D. Yang, T. Yan, Y. X. Song, M. Luo, “Hydrogen-bond-directed modular assembly of polar chains for rational construction of UV nonlinear optical crystals” *Angew. Chem. Int. Ed.* **64**, (2025): e202512342.

[44] Z. Q. Chen, C. X. Li, X. Y. Wu, J. J. Lu, Z. H. Yang, X. L. Hou, M. R. D. Mutailipu, “Engineered integration of diverse functional units induces multifunctionality in malonate-borate hybrids” *Aggregate* **6**, (2025): e70134.

[45] Z. Y. Lin, H. L. Liu, L. L. Cao, L. Huang, X. H. Dong, Y. Q. Zhou, D. J. Gao, N. Ye, “Deprotonation-regulated conformational switching in malonates enables high-performance ultraviolet polar crystals” *Inorg. Chem.* **64**, (2025): 20832–20841.
